# Age-associated changes in the circulating human antibody repertoire are upregulated in autoimmunity

**DOI:** 10.1186/s12979-020-00193-x

**Published:** 2020-10-06

**Authors:** Aaron Arvey, Michael Rowe, Joseph Barten Legutki, Gang An, Anantha Gollapudi, Anna Lei, Bill Colston, Chaim Putterman, David Smith, Janelle Stiles, Theodore Tarasow, Preveen Ramamoorthy

**Affiliations:** 1 iCarbonX 2424 Camino Ramon, Suite 125, San Ramon, CA 94583 USA; 2 HealthTell, 145 S. 79th St., Chandler, AZ 85226 USA; 3 Albert Einstein College of Medicine, Division of Rheumatology, Forchheimer 701N, 1300 Morris Park Ave, Bronx, NY 10461 USA; 4 Azrieli Faculty of Medicine, Bar-Ilan University, Zefat, Israel; 5 Research Institute, Galilee Medical Center, Nahariya, Israel

**Keywords:** Antibody binding profile, Immune age, Auto-immune disease, Machine learning, Immunosenescence, Antibody response, Peptide library

## Abstract

**Background:**

The immune system undergoes a myriad of changes with age. While it is known that antibody-secreting plasma and long-lived memory B cells change with age, it remains unclear how the binding profile of the circulating antibody repertoire is impacted.

**Results:**

To understand humoral immunity changes with respect to age, we characterized serum antibody binding to high density peptide microarrays in a diverse cohort of 1675 donors. We discovered thousands of peptides that bind antibodies in age-dependent fashion, many of which contain di-serine motifs. Peptide binding profiles were aggregated into an “immune age” by a machine learning regression model that was highly correlated with chronological age. Applying this regression model to previously-unobserved donors, we found that a donor’s predicted immune age is longitudinally consistent over years, suggesting it could be a robust long-term biomarker of humoral immune ageing. Finally, we assayed serum from donors with autoimmune disease and found a significant association between “accelerated immune ageing” and autoimmune disease activity.

**Conclusions:**

The circulating antibody repertoire has increased binding to thousands of di-serine peptide containing peptides in older donors, which can be represented as an immune age. Increased immune age is associated with autoimmune disease, acute inflammatory disease severity, and may be a broadly relevant biomarker of immune function in health, disease, and therapeutic intervention.

## Background

Ageing is associated with broad decline in organ function and increased risk for chronic disease. The immune system undergoes dramatic changes associated with age, including decreased immune response, loss of immune memory, and increased chronic inflammation. These immune dysfunctions manifest as re-activation of latent infection, decreased tumor immunosurveillance, and age-associated chronic immunopathologies [1–4]. Both adaptive and innate immune mechanisms are impaired, as evidenced by antigen-independent decreases in cellular proliferation and function [5, 6], migration [7], T-cell receptor diversity [8], antibody secretion [9], phagocytic abilities [10], cytotoxicity [11], and broad dysregulation of cytokines and chemokines [6, 12].

Ageing broadly impacts humoral immunity, as antibody affinity and the adaptive immune processes that lead to their production suffer with age [5, 13, 14]. For instance, plasma cells produce less antibody [15], germinal center B cell selection results in lower affinity antibodies in mouse [16], and the CD4+ T cell receptor diversity decreases [17]. Additionally, hematopoiesis broadly declines [4, 18–21], professional antigen presenting cells reduce expression of peptide-MHC-II complex [22, 23], and antibody effector cells show decreased functional clearance of IgG-bound pathogens [12, 24]. These age-dependent declines in humoral immunity can be manifested in less effective antibody binding [25, 26], which can result in differential infection protection as demonstrated by serum transfer experiments of heterochronic mice [27]. Mouse studies have further demonstrated that while antibody quality and quantity suffer with age, there is also a concomitant decreased specificity to foreign antigen and increased production of autoantibodies [28]. IgM autoantibody secretion is selectively induced in older mice in response to vaccination, whereas unvaccinated aged mice in semi-sterile lab environment presented with fewer self-reactive secreting splenic B cells [29].

While it has long been known that human antibody production is altered with age [30], which can lead to increased self-reactivity [31], more recent data suggests deeper links to autoimmune disease etiology and impact on broader metrics of quality of life. B-cell diversity from donors > 86 years old vs those < 54 years can be dramatically reduced, which is then subsequently correlated with measurements of frailty, survival, and vitamin deficiency [13].

To better understand and quantify the impact of ageing on the immune response, we identified age-associated patterns in serum antibody binding profiles. We profiled IgG antibody binding using peptide microarrays in a cohort of 1675 donors. We created a machine learning model that estimates an “immune age” from a donor’s antibody binding profile that is highly correlated with chronological age. The immune age is highly robust with respect to technical parameters, such as reagents, peptide microarray design, and serum handling. The machine learning regression model was validated on an independent donor cohort and longitudinal profiling revealed that a donor’s immune age is typically consistent over multiple years suggesting that this could be a robust long-term biomarker of age-associated humoral immune decline. We show that accelerated immune ageing, when a donor has an older immune age than chronological age, is associated with autoimmunity, autoinflammatory disease, and acute disease flares. These results suggest that the immune age may be a broadly relevant biomarker of immune function in health and disease.

## Results

### Profiling the circulating antibody repertoire in a demographically-diverse cohort

**Fig. 1:**
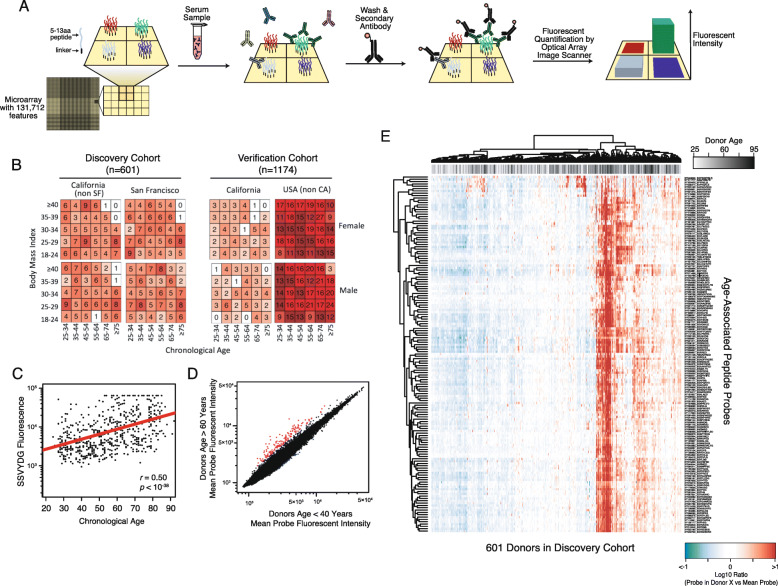
Antibodies isolated from human sera show different binding profiles in older compared to younger donors. **a** Peptide arrays were manufactured with over 131 k diverse probes to assess IgG antibody binding. The assay workflow includes incubating donor serum sample on the peptide microarray, detecting bound IgG with a fluorophore-conjugated secondary antibody, and quantifying the fluorescent signal at each feature. A subset of four peptide features are shown along with cognate binding antibody molecules (as indicated by color). **b** The donor cohorts were designed to obtain diverse sampling of donor demographics, including age, BMI, sex, and geography from multisite recruitment. Combinations of age and BMI were explicitly balanced, as were other combinations of demographics. **c** Age (x-axis) is highly correlated with many probes’ fluorescent intensities; for example, probe XY064981 with peptide sequence SSVYDG (y-axis) fluorescent intensity and age across *N* = 601 donors (each datapoint) has Pearson’s correlation coefficient of *r* = 0.50 (*p* < 10^− 38^). **d** There are 100 s of peptide features that are significantly associated with older vs. younger serum donors (red points). The average peptide intensity of younger donors (x-axis) versus older donors (y-axis) shows the differential expression of all peptides. Every data point is a single peptide probe on the array. Alternative estimates of effect size and significance yield similar results (Figure S1). **e** Probes associated with age are highly correlated: if one age-associated peptide probe has elevated fluorescent intensity in a given donor, it is likely that fluorescence of many age-associated peptides are increased. Age-associated probes (y-axis, selected red points in Fig. 1d) are shown across all 601 donors in the cohort (x-axis). Donors are labeled by age (gray-scale legend) and probe intensities values are shown as Log10 ratio of probe in specific donor versus mean probe intensity across all donors. Hierarchical clustering was performed on donors and probes independently

To understand antibody binding distributions in healthy donors, we quantified antibody binding in a large demographically diverse cohort (Fig. 1a, Figure S1A). Antibody-peptide binding was measured by diluting serum samples, incubating on peptide arrays, labeling bound antibodies using fluorophore-conjugated secondary anti-IgG antibody, and imaging the arrays to quantify fluorescent intensities (Fig. 1a, Methods). High-density peptide microarrays were synthesized with ~ 125,000 distinct, untargeted peptide sequences as previously described (Methods). Previous studies using the same array design were able to predict chronic infections [32]. This approach enabled us to profile a broad sample of antibodies present in each serum sample.

Our demographically diverse cohort was enrolled prospectively at multiple collection sites that obtained venipuncture donor samples and collected self-reported age, weight, height, and sex (Methods). To minimize bias associated with self-selecting blood donors, we pre-specified a balanced donor enrollment by age, sex, and geography (Fig. 1b). In total, 1675 samples were obtained for training and verifying conclusions. A “discovery” cohort was recruited July-Sept 2017, resulting in 601 donor serum samples. A “verification” cohort was then recruited from a distinct set of donors Sept-November 2017, obtaining samples from 1074 donors. All studies were performed with Institutional Review Board approval (Methods).

### Chronological age is highly correlated with serum antibody binding profiles

We assayed 601 donor samples to measure antibody binding profiles in a healthy donor population (discovery cohort). Pearson’s correlation analysis of chronological age revealed thousands of peptides with statistically significant correlation (Fig. 1c-d, Figure S1B-D). Effect size for age was estimated using the age coefficient of linear regression and log-ratio of average peptide fluorescence of older (> 60 years) compared to younger (< 40 years) donors (Figure S1D). The thresholds of 40 and 60 years were selected to balance sample size, donor demography diversity, and diverse probe effect size. When we selected the highest effect size probes (each having log10 ratio > 0.15 and Bonferroni-corrected statistical significance *P*_*FWER*_ < 0.01), we found that if older donor serum differentially bound any of these peptides (with log10 ratio > 0.15) that it was likely to bind many other age-associated peptides (Fig. 1e). This intra-donor correlation of the most age-associated probes suggested that a common peptide sequence motif may be driving antibody-peptide binding.

The age-associated probes were highly reproducible in technical replication experiments that used array manufacturing reagent lots independent from the initial assay. We confirmed results by taking a subset of 66 samples and re-assaying them to confirm similar values. For this technical replicate cohort, we selected donors that did and did not bind highly to age-associated probes and were young and old. This 2 × 2 selection criteria enabled us to determine if the age-associated probes were stochastically bound tending towards age-specific binding or if they were consistent for a given sample, irrespective of age. The technical replication cohort confirmed that the same probes were bound (Figure S2A-C). Importantly this analysis also confirmed that irrespective of age, the binding patterns of a given donor to age-associated probes was technically reproducible (Figure S2D).

### Antibodies from older donors bind peptides with an N-terminus di-serine motif

**Fig. 2:**
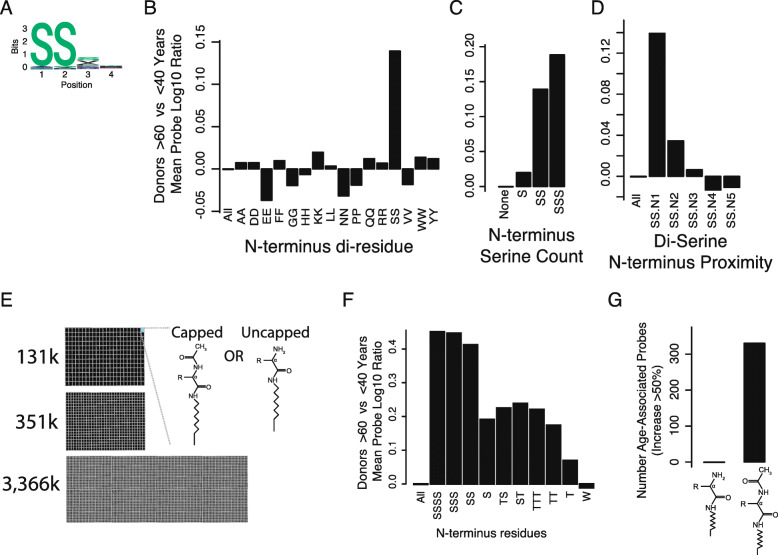
Peptide sequence motifs in probes associated with age. **a** Sequence motifs in peptide probes associated with age. Peptides associated with age contain a strong N-terminus di-serine (N-di-serine) motif. Motif information content (bits, y-axis) is shown for each position (x-axis). **b** The N-terminus di-serine motif is much more associated with age (y-axis) than any other di-residue motif (x-axis). **c** The number of serine residues at the N-terminus (x-axis) is correlated with age-associated antibody binding (y-axis). **d** Age-associated peptide binding decreases with increased distance of di-serine from N-terminus. The starting position of di-serine residues (x-axis) relative to the N-terminus. The N-terminus is defined as *N* = 1. **e** To further characterize the peptide motifs, multiple peptide array synthesis modalities were employed (see Methods). Arrays with ~ 131 k, ~ 351 k, and ~ 3366 k probes were synthesized with peptides that had N-terminus acetyl-capping, a free N-terminus amine, or contained probes with both capped and free N-termini. **f** Older and younger donor sera were assayed on large microarray format with 3366 k non-control probes, which contained a broader set of peptide probes and inclusion of amino acids, including threonine and isoleucine, which were excluded in the 131 k probe microarray. The presence of multiple N-terminus serines remains the most highly significant motif, and additional serines in positions 3 and 4 may increase discrimination slightly (*N* = 142 probes starting with tetra-serine). Motifs including N-terminus threonine, which is biochemically similar to serine, are the second-most associated motif. Tryptophan, which is typically the ‘stickiest’ amino acid due to the aromatic indole sidechain, is shown as a negative control that is not associated with age. **g** Age-associated antibody binding to the di-serine N-terminus motif requires that the N-terminus be acetylated. On arrays where both acetylated and un-acetylated (uncapped free amine) probes are on present on each individual microarray, only acetylated “SS” features show age-associated binding. The number of age-associated probes with > 50% increased binding in donors > 60 yrs. vs < 40 yrs. (y-axis) is shown for uncapped free-amine probes (left) and acetyl-capped probes (right). The cutoff of 50% is representative and other cutoffs can be found in supplemental material (Figure S3G)

The peptide sequence of probes associated with age contained a common pattern of serine residues at the array surface-distal N-terminus (Fig. 2a and Figure S3A). Of the largest effect size probes, > 90% had two consecutive serine residues at the N-terminus (N-di-serine motif, Figure S3D). The remaining < 10% probes started with a serine residue in one of the two residues at the N-terminus. The N-di-serine pattern was highly statistically significant (*P <* 10^− 41^; hypergeometric test) compared to other N-terminal amino acid dimers (Fig. 2b). Correlation to age increases with the number of N-terminal serines (Fig. 2c) and decreases as the di-serine is located further from the N-terminus (Fig. 2d). Due to manufacturing limitations of the standard array format, we could not expand on the di-serine motif since there were only 438 peptides on the array with an N-terminus di-serine.

To refine and better understand the age-associated binding to serine, we developed a peptide microarray synthesis that packed peptides more densely on a larger physical array (Methods). This new large-format array had 3366 k probes, which contained peptide sequences that tiled all peptide subsequences of length 5 (5-mers), many infectious disease proteins and antigens, autoimmune antigens, and about 4000 extra-cellular or secreted human proteins. Notably, the larger format array contains 16,092 probes that start with a N-di-serine motif, allowing us to better-characterize the adjacent residues that may influence antibody-peptide binding.

We selected a subset of samples to assay on the larger peptide array platform, based on N-terminus serine motif score and age, using same selection strategy as for technical replication cohort (*N* = 32). Again, we observed that nearly all age-associated probes contain a strong N-di-serine motif. While not all N-di-serine probes are statistically significantly age-associated, > 98% of N-di-serine probes have > 0 Pearson’s correlation with age suggesting that the vast majority of N-di-serine probes may be associated with donor age with a properly powered cohort. Due to the increased number and diversity of probes, the larger array format enabled the discovery of several sequences highly enriched in the top age-associated probes, including N-terminus motifs SS [VF]. However, these expanded motifs were only modestly statistically significantly more enriched than a homopolymer-serine motif.

To further expand the N-di-serine motif, we synthesized the larger format peptide array labeling every probe with N-di-serine, followed by the original peptide (Fig. 2e). This allowed us to exploit similar manufacturing protocol and synthesis controls while fixing an N-terminus di-serine and allowing probes to differentiate exclusively on non-N-di-serine influences on peptide-antibody binding. We discovered multiple statistically significant motifs; however, most significant was strikingly N-tetra-serine ”SSSS” (Fig. 2f). We also found that homo-threonine N-terminus motifs attracted increased antibody-binding, which was not discovered on smaller peptide array due to exclusion of threonine residues (Fig. 2f). More complex motifs, such SS[VF], had far less statistical significance and effect size.

Interestingly, an N-terminus acetyl-cap was required for antibodies to bind polyserine motifs (Fig. 2g). We synthesized both acetyl-capped and uncapped arrays, as we hypothesized that peptide charge may influence antibody-peptide binding. The acetyl-cap decreased overall peptide charge compared to arrays where the N-terminus was left as a free-amine. We then synthesized a single 351 k feature array that included two copies of the original 131 k peptide library, with one copy of the library being acetyl-capped and the other copy being uncapped. We found that only the acetyl-capped SS-peptides were bound preferentially in older donors (Fig. 2g). This 351 k-feature array enabled comparison of peptides that were side-by-side on a single array, which mitigated possible batch effects by synthesizing peptides simultaneously and assaying together on single array.

### Creating a N-terminus di-serine age-association score

Since the N-di-serine motif was prominently enriched in age-associated peptide probes, which had high intra-donor correlation, we calculated the average normalized fluorescent intensity of the age-associated N-di-serine containing probes (Methods). This simple aggregate statistic was remarkably robust across experimental assay conditions and peptide microarray format (Figure S3F).

**Fig. 3:**
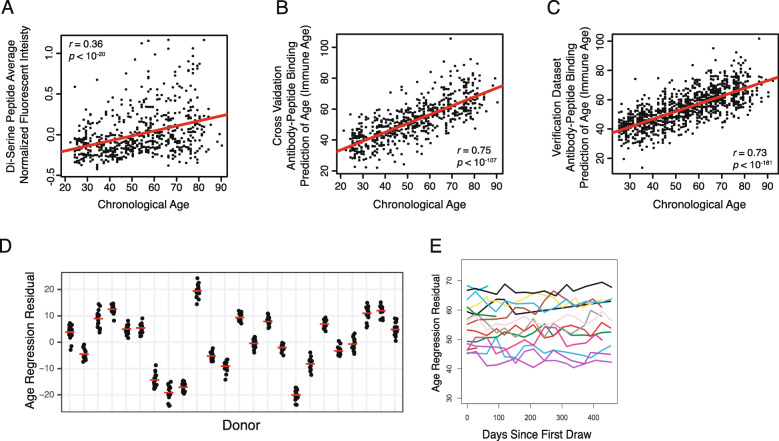
Antibody-peptide binding profiles are able to predict chronological age with high accuracy. **a** While the average N-di-serine probe intensity (y-axis) is highly associated with age (x-axis), the average normalized fluorescent intensity of age-associated N-di-serine probes is only moderately predictive for chronological age (Pearson’s *r* = 0.36). **b** An elastic net regression model of peptide array probe intensity data is able to predict chronological age with high accuracy on holdout examples during model training. Each data point is a single donor, showing the age of donor (x-axis) and prediction of age based on regression model of antibody binding profile (y-axis). Pearson’s correlation coefficient of *r* = 0.75. **c** The model learned from the Training Cohort is applied to the Verification Cohort. Pearson’s correlation coefficient is *r* = 0.74 (*p* < 10^− 181^, 95% confidence interval of [0.71, 0.76]). **d** The age regression residuals (y-axis) for 24 Donors (x-axis) are highly reproducible. Each donor was assayed in 16 technical replicates, which were balanced across multiple days, array manufacturing synthesis lots, secondary antibody reagent lots, and sample dilution aliquots (Methods). Each data point is a single assay for a single donor. **e** The age regression residual values (y-axis) are consistent across *N* = 16 donors that consented to regular blood draws for > 1 yr. Donors with > 5 samples over > 1 yr (*N* = 13) had consistent age-regression values over this time period. Data shown for all donors (lines, color indicates donor)

While the N-terminus di-serine motif was strongly enriched and statistically significant, it was only partially predictive of chronological age. Many older donors had limited antibody binding for probes with N-terminus di-serine and a subset of young donors presented with high binding to these peptides. There was high enrichment of highly bound N-di-serine probes in donors > 60 years vs < 40 years (probes that were > 1.8 fold higher than array-median; odds ratio of 7.3; two-sided Fisher’s exact test). However, Pearson’s and Spearman’s correlation coefficients between this score and chronological age was low (*r* = 0.36 and *ρ* = 0.35; *p* < 10^− 20^; Fig. 3a). Thus, the N-terminus di-serine motif suggests potential biological mechanism underlying age-associated antibody binding shift, but may not be sufficiently predictive of chronological age to act as an age-related predictive biomarker.

### Machine learning model of serum antibody binding predicts chronological age

We hypothesized that a predictive immune age could be developed with antibody binding profiles. This predictive score could then be compared to chronological age to estimate “accelerated ageing” of the antibody repertoire, or immune age. Similar “biological age” metrics based on cell counts, gene expression, cytokine expression, blood and leukocyte epigenetics, telomere length, and genetic predispositions thereof have been found to be predictive of health, disease, and even all-cause mortality [33–41], suggesting that molecular correlates of age can be useful biomarkers.

To develop an antibody repertoire immune age, we used the antibody binding dataset from the prospectively collected demographically diverse cohort of 1675 donors. These donors were acquired in two phases, with the first 601 collected being used as a “discovery” cohort and the subsequent 1074 used as verification. To obtain an initial estimate of antibody profile prediction of age, we performed 10-fold cross validation on the discovery cohort. While several machine learning methods were characterized, the elastic net [42] yielded an interpretable linear regression model that could be well regularized and easily applied to new unseen data. The age predictions for each serum sample were calculated from the cross-validation fold in which the example was in the test set.

Our machine learning regression model was highly predictive of chronological age in the hold-out folds (Pearson’s correlation coefficient *r =* 0.75*, P* < 10^− 107^; Fig. 3b). This strongly suggested that serum antibody affinities change during ageing and that older donor sera can be identified by peptide microarray fluorescent intensity.

We confirmed the accuracy of this model on a prospectively collected, independently recruited cohort of 1074 donors. This cohort was enrolled in a non-overlapping time interval as the 601 member Discovery Cohort and was sampled from greater geographical diversity across many venipuncture-collection sites (across the USA, whereas Discovery Cohort was sampled from California sites). The samples were assayed on independently manufactured peptide arrays, which were synthesized months after the original peptide arrays.

We confirmed that the machine learning regression model was highly predictive of chronological age in the Verification Cohort of 1074 donors (Pearson’s correlation coefficient *r =* 0.73, *P* < 10^− 181^; Fig. 3c). We also assayed independently collected samples from a myriad of other prospectively collected and banked samples, where we found similar accuracy in control populations of autoimmunity, infectious disease, cancer, and immunodeficiency case-control studies (data not shown).

### Desired characteristics of an immune age metric

In addition to being highly correlated with chronological age, any biological age should also satisfy additional constraints: (1) the biological age representing accelerated ageing should be statistically robust across resampled training sets and machine learning models; (2) biological age should be consistent across different assay modalities since the biological age should be specific to a donor, not due to experimental variation; (3) the biological age should have less variation within repeated measures of a donor than the variation between donors; and (4) longitudinal samplings should have relatively low variance with changes trending at a similar rate as chronological age.

### The immune age is statistically robust

We hypothesized that if a donor’s immune age was higher than their chronological age, the “accelerated” ageing of the humoral immune response may be associated with immunopathology or broader disease physiology (Figure S4). For the immune age to be a relevant biomarker of disease or risk thereof, it must be highly reproducible. Furthermore, since we hypothesize that the differene between immune age and chronological age is the relevant metric, then this regression residual must be highly reproducible. Typically, residuals are modeled as randomly distributed noise, e.g., in the model *y* = *xβ* + *N*(0, *ϵ*), which can suggest that residuals are in fact stochastic or experimental noise; however, non-stochastic residuals may suggest a true deviation of antibody profile from the expected given chronological age (Figure S4). This could be evidenced by reproducible immune age across a number of permutations of training set data, machine learning algorithm, repeated assay, different assay modalities, and similar statistical considerations.

To increase the likelihood of immune age being statistically robust, we quantified residuals across models on a shared hold-out set and found that regression residuals are statistically robust. We selected machine learning parameters that yielded statistically higher bias and lower variance (after decomposing the sum of squared residuals into average bias and variance, Figure S6A, see Methods). Combining the two cohorts together, we optimized the machine learning model by testing sparsity constraints and regularization parameter impact on the bias and variance (Figure S6A). From the combined cohort of size 1675 donors, we sampled two mutually exclusive training sets and one test set. The models learned from the two training sets were compared on the one test set to determine accuracy, bias, and variance. This process was repeated 100 times and results suggested a semi-sparse classifier including ~ 5–10% of features be included in a final model that had lower variance and higher bias, while achieving near optimal accuracy (Figure S6B). The immune age, as predicted using the optimized machine learning model on a two independent training sets, had a high Pearson’s correlation coefficient on a single shared test set. This suggests that machine learning model and training set sampling-induced variance is low (Figure S5A).

### Immune age residuals are robust across technical replicates

We also confirmed that residuals were broadly consistent across diverse training and hold-out datasets. When we trained on divergent peptide arrays that were synthesized using different strategies (e.g., N-terminus acetylation), we found the immune age predicted on a held-out test set were comparable (Figure S5A). Similarly, if we used a different machine learning model (e.g., support vector regression in place of the elastic net), we obtained similar residuals. We confirmed that assay reagent lots and peptide arrays synthesized across a 20 month window (April 2017 – January 2019) yielded comparable the immune ages, further confirming that the immune ages are technically reproducible (Figure S5B). Regression analysis performed on donors binned by ethnicity and sample collection site yielded predictions with similar correlation to age (Figure S5C) that were not significantly different in an ANOVA (Figure S5D). Finally, we used completely different peptide feature libraries, including arrays with 131 k, 351 k, and 3366 k distinct probes, where we were able to examine residuals for the same sample when machine learning models were developed and verified using different peptide sequences on different peptide microarray platforms (Figure S5B).

This confirmed that immune ages were robust to exact choice of machine learning model, training dataset, assay batches, array synthesis procedures, and shared test sets. The robustness of residuals across these diverse technical variations (and longitudinal stability, described below) strongly supports a donor-specific residual that is non-noise, non-stochastic, and may be biologically relevant.

### Immune age intra-donor variation is less than inter-donor variation

To further confirm that antibody binding regression age-residuals were not experimentally stochastic, we selected 24 donors to assay in 16 replicates each. These donors were selected to ensure reproducibility across a large dynamic range (Methods). The age residuals were highly reproducible, presenting with much lower intra-donor variation than inter-donor (Fig. 3d). Average standard deviation was +/− 3.7 years, which is < 10% of the total range of 40 years. Furthermore, donors at the extreme ends of the dynamic range distribution presented with homoscedastic variance. This strongly supports a donor-specific residual that is not driven by batch or replication noise. Formal analytic validation of this assay will be described elsewhere.

### Age-associated antibody binding is stable in vivo for > 15 months

We recruited a cohort of healthy donors to have regular blood draws on an approximately bi-monthly basis and assayed antibody binding via peptide microarray (Methods). We found that donors had an average standard deviation of +/− 5.2 years. Since the age range of the donors profiled was ~ 40–70 years, the standard deviation is ~ 15% of total range (Fig. 3e). We found similar stability of the SS-score, which was also < 10% variation of range (Figure S5E).

### Prediction of chronological age is not improved by cytokine concentrations

We hypothesized that the addition of serum cytokine concentrations to the antibody binding data would improve prediction of age. A custom panel of cytokines was measured and a regression model built (Figure S7). We found that immune age predicted from cytokine and antibody binding generally agreed, but did not enhance prediction when combined.

### Age-associated antibody-peptide binding is not impacted by endogenous small molecules, common exogenous interferents, nor detection reagents

While IgG is a highly abundant serum-protein, there are many small molecules that are present in much greater concentration. The binding of IgG molecules to arrayed peptides could potentially be impacted by interfering molecules that are also correlated with age. Since our goal is to discover antibody-mediated mechanisms of the ageing immune system, we wanted to ensure that the peptide microarray platform was directly assaying peptide-antibody interactions based on direct antibody binding for the peptide sequence.

**Fig. 4:**
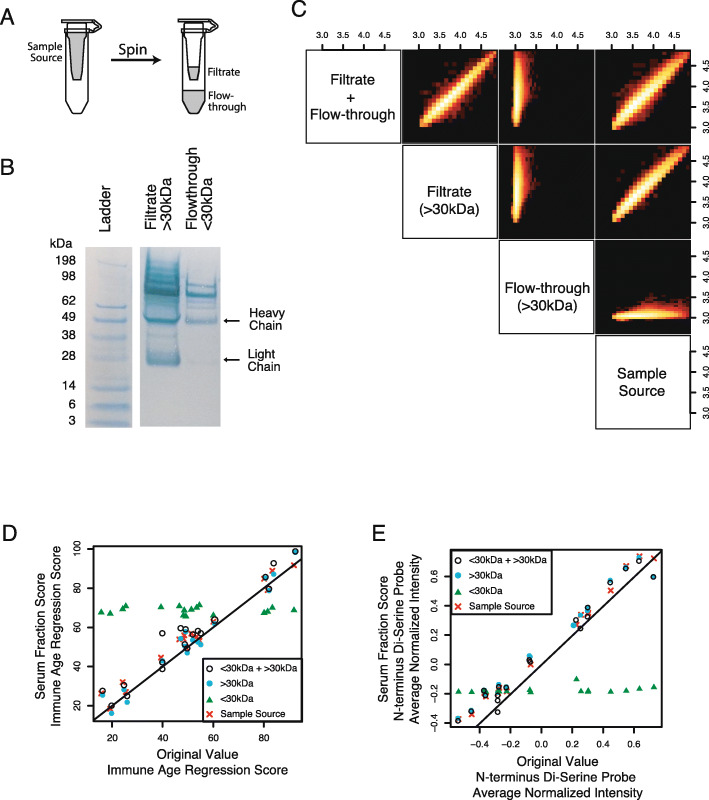
Serum antibodies are required for predicting chronological age from peptide array binding data. Furthermore, serum small molecules do not contribute to prediction of chronological age. **a** Schematic of column size filter. The 30 kDa filter columns can be used to separate serum molecules into flow-through fraction that contains < 30 kDa molecules and filtered fraction that contains > 30 kDa molecules. **b** Size filter columns are effective at depleting IgG using a 30 kDa filter, as quantified by Coomassie Blue staining. Filtrate (> 30 kDa) produces bright bands for both light and heavy chains. Flow-through (< 30 kDa) is depleted for heavy and light chain; however lower concentrations of > 30 kDa molecules can still be seen. Ladder standard and heavy/light chain weights are annotated. Image is crop edited and rotated, unedited image can be found in Figure S8. **c-e** Antibody purification through column filter shows that IgG is required for prediction of chronological age. Sixteen donor samples were selected to obtain coverage of chronological age regression dynamic range (Methods). These 16 samples were processed in 4 ways: (1) no processing (sample source), (2) filtered through 30 kDa column and only the filtrate (> ~ 15 kDa molecules retained; filtrate), (3) filtered through 30 kDa column and only the flow-through retained (<~ 75 kDa molecules retained; flow through), and (4) the filtrate and flow through were recombined after running through column. **c** Correlation between log10 peptide intensities show sample source, filtrate + flow-through, and filtrate all recapitulate original signal. In contrast, the flow-through alone, which is IgG depleted, has no correlation with original peptide-antibody binding. **d** In addition to raw signal being recapitulated, the machine learning regression model is recapitulated only when IgG is present. The 16 samples are plotted as machine learning regression values from the original (x-axis) and filter column-processed (y-axis). **e** Same as (**d**), but axes’ values are the di-serine peptide score rather than chronological age regression model

To determine whether a non-antibody serum factor may be altering antibody binding, we performed serum fractionation studies to enrich/deplete specific fractions for IgG and/or other molecules. The most definitive fractionation was performed by size column concentration (Fig. 4a). Columns that used 30 kDa filters were able to significantly deplete antibody heavy and light chains in the eluent while concentrating these and other large protein molecules in the filtrate (Fig. 4b). We confirmed that the eluent fraction (depleted for IgG) had very limited peptide-array fluorescent signal, whereas the filtrate containing IgG was highly concordant with original serum samples pre-fractionation (Fig. 4c).

The antibody binding regression as performed on the original sample was re-capitulated on the re-assayed sample, the recombined fractions, and filtrate, but not the eluent (Fig. 4d). Similarly, the N-terminus di-serine intensities were reproduced only with the fractions containing IgG, with no signal found from IgG-depleted fractions (Fig. 4e).

We also examined probes that typically bind labelled secondary antibody directly and found that they were not differentially bound in older vs. younger donor serum samples (Figure S8B) and that common immunoassay interferants do not produce a signal that is similar to that observed for younger-vs-older serum antibody binding (Figure S8C,D).

### Autoimmune phenotypes are associated with an accelerated immune age

Since age-related humoral immune decline is associated with decreased antibody binding for pathogens and increased frequency of autoantibody generation [25–29, 31], we characterized our antibody binding regression model in a cohort of autoimmune and phenotypically similar diseases. We enrolled cohorts of non-autoimmune diseases (fibromyalgia, osteoarthritis, vascular disease, and similar diseases) and autoimmune diseases, such as Sjogren’s syndrome, rheumatoid arthritis (RA), and systemic lupus erythematosus (SLE). For most donors, we had longitudinal acquisition of serum samples over > 1 yr. We also obtained metadata regarding disease activity and molecular assays (e.g., anti-dsDNA autoantibodies). While performing antibody binding profiling by peptide microarray, we balanced diseases and disease activity (where known) between assay batches.

**Fig. 5:**
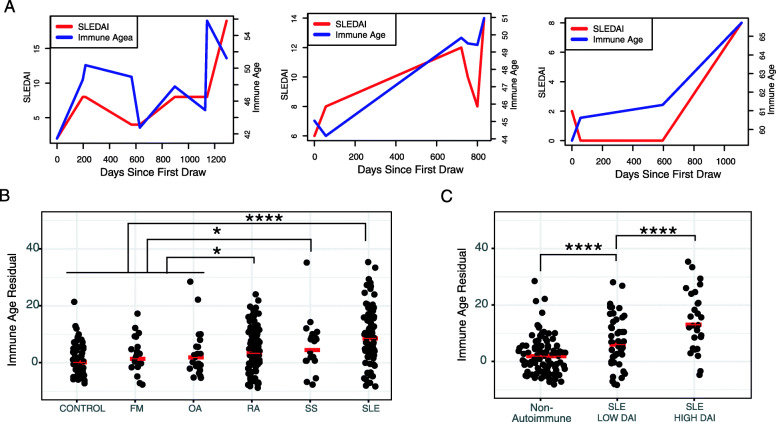
Donors with autoimmune disease have “accelerated immune ageing” as quantified by antibody binding profiles associated with higher age than subject’s chronological age at blood draw. **a** Longitudinal profiling of the antibody repertoire is correlated with disease activity index in donors with systemic lupus erythematosus (SLE-DAI). SLEDAI and Immune Age are shown (y-axis) relative to days since first visit (x-axis) for three donors (distinct plots). When the maximum disease activity is compared to lowest disease activity for each donor, we find that the Immune Index is higher when SLEDAI is higher (*p* < 0.04, paired t-test). **b** Age regression residuals are higher in serum from donors with autoimmune diseases. Donors with autoimmune, autoinflammatory, and phenotypically similar diseases were profiled by peptide microarray and antibody-binding prediction of age was calculated. Donors with autoimmune disease had higher antibody-based prediction of age (after correction for chronological age) than healthy control donors and donors with phenotypically similar non-autoimmune diseases. Significance was determined by a two-sided t-test comparing non-autoimmune to SLE (*p* < 10^− 9^), RA (*p* < 10^− 5^), SS (*p* < 10^− 3^). Non-autoimmune diseases included fibromyalgia (FM), osteoarthritis (OA), vascular disease (VASC), and other diseases (data not shown). Autoimmune disease profiled were Sjogren’s syndrome (SS), rheumatoid arthritis (RA), and systemic lupus erythematosus (SLE). **c** Donors with high autoimmune disease activity in systemic lupus erythematosus (as measured by SLEDAI), have higher age regression residuals, which suggests SLEDAI is associated with accelerated antibody binding ageing. The SLE cohort was discretized into donors that had high disease activity (> 5 SLEDAI) vs low disease activity (<=5 SLEDAI). When multiple samples were available for a given donor, the sample with highest SLEDAI was used. Donors with non-autoimmune disease had lower antibody-binding age predictions than low SLEDAI donors (*p* < 10^− 3^, two-sided t-test), who in turn had lower age predictions than high SLEDAI donors (*p* < 10^− 5^, two-sided t-test)

In general, immune age values calculated in this cohort varied little between longitudinal samples from the same donor, consistent with previous observations. However, the range of the immune age in a subset of the autoimmune cohort was much greater. Specifically, across longitudinally samples from the same donor, higher SLE disease activity (as measured by SLEDAI (Systemic Lupus Erythematosus Disease Activity) score) was associated with accelerated ageing (Fig. 5a). More broadly, comparing autoimmune cases to healthy controls and non-autoimmune phenotypically similar diseases revealed a striking increase in antibody binding residuals (Fig. 5b).

These findings were further confirmed by analyzing patients with SLE that had high vs low disease activity. We selected a single time point for each donor that was annotated with that individual donor’s maximum SLEDAI score. We then compared these maximum SLEDAI scores to find donors that obtained high (> 5) vs. low (< 5) SLEDAI. Healthy donors and those with non-autoimmune disease had lower antibody-binding age residuals than donors with low SLEDAI, who in turn had lower age residuals than donors with high SLEDAI (*p* < 10^− 3^; *p* < 10^− 5^, respectively; two-sided t-test; Fig. 5c).

## Discussion

We have discovered an relationship between chronological age and binding of serum antibodies to a specific set of peptide sequences. We trained a robust machine learning regression model to estimate immune age that performed well on an independent prospectively recruited verification cohort. Furthermore, autoimmune disease and SLE disease activity were both associated with increases in immune age relative to chronological age. This constitutes proof-of-concept for using antibody binding directly as a biomarker in complex autoimmune diseases.

Antibody binding to short peptide motifs, such as the age-associated N-di-serine motif, may augment autoantibody formation. For instance, the Fc fraction of IgG, which rheumatoid factor targets, contains multiple ‘SSV’ motifs, which we also observed in peptides preferentially bound in older donors. While rheumatoid factor doesn’t bind these motifs in co-crystals with IgG Fc (primarily binding to CH2 and CH3 domains) [43, 44], it is intriguing that multiple di-serine motifs may stabilize interaction to enhance autoantibody avidity.

We demonstrated the biological relevance of the immune age in autoimmunity; however, other biological and immune-related ageing studies have found broader implications of dysregulated ageing processes. For instance, combinations of inflammatory markers (CRP, IL-6, IL1β, sTNFAR1) can predict long-term cardiovascular risk and all-cause mortality in older adults [38, 39]. Other biological age markers, such as telomere length, the epigenetic clock, and immune gene expression are associated with disease and all-cause mortality [35–37, 40], but neither provide a clinic-ready marker that specifies which physiological processes are awry. Performing large scale studies to characterize blood-based biomarkers of ageing is becoming more tractable with resources such as the UK Biobank enabling a 5-year study of mortality in 498,103 donors [34].

The peptide microarray technology is well-suited to use in large clinical studies, as it can be mass produced and antibodies remain stable in frozen serum for months. This contrasts with flow cytometry, which is an effective way to quantify age-associated segregation of cell subpopulations based on cell surface markers [40], but challenging to implement outside of the research environment due to sample instability, cold-chain requirements, assay cost, and large batch effects leading to inconsistent datasets.

Prophylactic interventions that reduce the immune age in a healthy population may lead to improved health outcomes. A recent study suggested that therapeutic interventions could reverse epigenetic proxies for biological age [45]. Therapeutically targeting reversal of immunosenescent trends [46, 47], plasma cell antibody secretion, and chronic inflammation [39] is possible and may be directly linked to or assayed by antibody binding profiles. Non-pharmacological interventions may be promising for reducing the immune age in a healthy population. For instance, vitamin E supplementation improves CD4+ T cell synapse formation and exercise is broadly beneficial to both human and mouse immune responses [48].

## Conclusions

The circulating antibody repertoire has increased binding to thousands of di-serine peptide containing peptides in older donors, which can be represented as an immune age. Increased immune age is associated with autoimmune disease, acute inflammatory disease severity, and may be a broadly relevant biomarker of immune function in health, disease, and therapeutic intervention. The immune age has the potential for wide-spread use in clinical and consumer settings.

## Methods

### Serum sample acquisition for population studies

Donor samples were obtained by venipuncture collected by United Blood Services (http://www.unitedbloodservices.org), and obtained from Creative Testing Solutions (CTS, Tempe, AZ). Samples tested negative for a panel of infectious diseases, including Hepatitis B Virus, Hepatitis C Virus, West Nile Virus, *T. cruzi*, and HIV. All samples were collected in the USA at diverse geography (Supp Figure S1A).

After receiving shipment of frozen 1–1.5 ml samples on dry ice, specimens were thawed and a portion of each was aliquoted into single use volumes and stored at − 80 °C. The remaining undiluted sample volume was stored at − 80 °C and re-aliquoted as necessary. Samples were tracked using 2D barcoded tubes (Micronic, Lelystad, the Netherlands).

### Human subject consent and annotation

All human subjects in this study consented to samples being used for research purposes. No test results were returned to donors. IRB oversight of the study was conducted: Western Institutional Review Board (protocol no. 20152816).

The following annotations were obtained for each donor: age, BMI, sex, ethnicity, and geographic location of original venipuncture blood donation. Except for location, all other annotations were self-reported. Age at time of blood donation was calculated by CTS from self-reported birthdate, which was not provided to protect donor privacy. BMI was calculated as weight (in kilograms) divided by squared height (in meters) in units of kg/m^2^. Sex was self-reported as male or female. Ethnicity was self-reported and then coarsely grouped into White, Latino, Asian, and Black. Site of blood donation was recorded and reported as San Francisco, Other California, Arizona, Nevada, California, North Dakota, Washington, Montana, and South Dakota, and Texas.

### Serum sample acquisition for longitudinal studies

A set of donors were longitudinally sampled an average of 8 times over an average of 385 days. Donor samples were obtained by venipuncture collected by trained phlebotomists. Samples were separated into serum, which was frozen and stored at − 80 °C.

### Peptide microarray synthesis (131 k)

Peptide microarrays were synthesized at a private facility in Chandler, Arizona, as has been previously described [32]. Briefly, each microarray contained 131,712 peptide features, each associated with a single peptide sequence and spatially randomly distributed. These features comprise two libraries: (1) a combinatorial library of 125,509 features used to estimate antibody binding and (2) a control library of 6203 features, which includes varying numbers of replicates of 542 peptides, including peptides with known binding to monoclonal antibodies, fiducial markers to aid grid alignment, analytic control sequences and surface-linker-only features. The amino acids methionine and cysteine amino acids were excluded due to their potential to oxidize or cyclize. Additionally, isoleucine and threonine were excluded because of their chemical and structural similarity to valine and serine, respectively. Impact of isoleucine-to-valine and threonine-to-serine substitutions on age-associated probes was examined on larger format arrays and similar age-association was found (3366 k and 351 k, data not shown here). Peptides had a median length of 9 residues, ranging from 5 to 13 amino acids in length. The peptide sequences included 99.9% of all possible 4-mers and 48.3% of all possible 5-mers of the 16 amino acids.

Peptides were synthesized on 200 mm (mm) silicon oxide wafers using semiconductor photolithography, as previously described [32]. Briefly, an aminosilane functionalized wafer was coated with BOC-glycine and a photoacid generator, which is activated by UV light. A set of photomasks were used to expose specific features on the wafer to UV light (365 nm). These masks were employed iteratively to add activated amino acids, some with protected side groups, to the N-terminus of peptides. At the end of final cycle, the N-terminus of the chain is capped by an acetyl group. Next, each wafer was diced into 13 slides of dimensions 25 mm × 75 mm containing 24 microarrays arranged in eight rows by three columns. Amino acid side chains were deprotected as previously described and slides stored in a dry nitrogen environment until assay.

Slides are grouped into gasket-partitioned cassettes, each of which holds 4 slides. Since each slide includes 24 independent arrays, this permits 96 samples to be assayed per cassette in a microtiter plate format.

### Design and synthesis of larger format peptide microarrays

351 k: Synthesized as above, except microarrays contain 351,909 total peptide features printed at higher density, and includes the amino acid threonine in some peptides. The 3366 k library contains two copies of the 131 k library, where one has an acetylated N-terminus and the other a free amine at the N-terminus. The 3366 k library also contains peptide features that represent known autoantigens and other hypothesis-driven probes.

3366 k: These arrays combine larger area with higher printing density to provide 3,366,522 peptide features. The present study focused on a combinatorial library of 1,889,568 unique octamer peptides that included all possible pentamers of 18 amino acids (the 131 k set plus threonine and isoleucine). Greater than 99% of the unique pentamers occur exactly once within some peptide at each of the four positions from the N-terminus. The design also includes 1,328,926 peptides tiled to known epitope or protein sequences from the literature, and 148,028 control features.

3366 k-SS: Following standard 3366 k array synthesis as described above two, additional cycles added di-serine to the N-terminus prior to N-terminal acetylation. A special mask was used to photo-expose all features on the array. Following photo-deprotection, serine was coupled to all features.

### Peptide microarray synthesis quality control

Batches of peptide microarrays were assayed by MALDI-MS to verify that peptide extension cycles incorporated the proper amino acids. From this, coupling efficiencies were calculated and found to be typically > 97% (with typical confidence interval of 95–100%, depending on cycle and amino acid pair). This suggests that for peptides of length 10, we expect > 70% of peptides to be correctly synthesized and the remaining 30% to include some amino acid deletions (usually no more than one). Wafer manufacturing was tracked from beginning to end in a relational database. Data typically tracked include chemicals, recipes, time and technician performing tasks. After a wafer was produced the data were reviewed and the records were locked and stored. Finally, each lot was evaluated in a standard binding assay and sample set to confirm performance.

### Antibody-peptide microarray binding assay

Aliquots of 20 μL serum were thawed on bench for 30 min. Post-thaw, samples were invert mixed and centrifuged. Samples were then diluted to 1:625 in 1% mannitol in PBST+P (phosphate buffered saline, 0.05% Tween 20, 0.1% Proclin 950) assay buffer (8 μL sample diluted into 4992 μL buffer). Sample is then vortexed. All aliquoting and dilution steps were performed using a BRAVO robotic pipetting station (Agilent, Santa Clara, CA). All procedures, which used de-identified, banked plasma samples, were reviewed by the Western Institutional Review Board (protocol no. 20152816).

Peptide microarray slides are assembled into 4-slide cassettes and the automated assay is performed by an integrated robotics system containing all necessary modules to process slides. Microarrays were rehydrated by soaking with distilled water for 1 h (h), PBS for 30 min (min) and primary incubation buffer (1% mannitol, PBST-P) for 1 h. Microarray slides were rinsed in distilled water to remove residual salts and centrifuged briefly to remove excess liquid. Samples were incubated on arrays for 1 h at 37 °C with mixing. Following incubation, the cassette was washed three times in PBST-P using microtiter plate washer (BioTek Instruments, Inc., Winooski, VT). Serum antibody binding to peptide features was detected using 4.0 nM goat anti-human IgG (H + L) conjugated to AlexaFluor 555 (Invitrogen-Thermo Fisher Scientific, Inc., Carlsbad, CA) in secondary incubation buffer (0.5% casein in PBST) for 1 h with mixing on a TeleShake95 platform mixer, at 37 °C. Following incubation with the secondary antibody, the slides were again washed with PBST-P, followed by distilled water. After removal from the cassette, the slides were sprayed with isopropanol and centrifuged dry. Quantitative signal measurements were obtained by determining a relative fluorescence value for each addressable peptide feature.

### Peptide microarray data acquisition

An ImageXpress imaging system was used to detect secondary anti-IgG antibody conjugated to AlexaFluor 555 or DyLight 550. The imager used an LED light engine (SemRock) centered at 532 nm wavelength to excite fluorophore-conjugated secondary antibody (ThermoFisher Scientific). We initially used the Mapix software application (version 7.2.1; Innopsys, Carbonne, France) to grid images into individual peptide intensities and developed custom image analysis software for the larger format arrays (3366 k and 3366 k-SS) where optical warping caused significant distortion of fluorescent signal (ImageTool software, described elsewhere). Median foreground pixel intensities for each peptide-feature were calculated in an using the central 60% of feature pixels, which allowed gridding in accuracy without catastrophic failure. Array scans were saved as TIFF images. Gridding output was saved to GenePix Result format files with peptide features taking values in the range ~ 500 to 65,535 Relative Fluorescence Units.

### Regression modeling of ageing impact on antibody binding

Two studies, labelled experiments 1068 and 1116, were used as discovery, feasibility, and verification datasets. In experiments 1068 and 1116, there were a total of 601 and 1074 samples, respectively, that were obtained from Creative Testing Solutions and assayed by HealthTell’s ImmunoSignature system. All samples were incubated on 131 k arrays following the above protocol. From this data, we were able to derive a regression model that could predict a donor’s age from peptide array data (Fig. 1).

The regression model was an Elastic Net learned from experiment 1068 with parameters tuned based on performance (correlation accuracy) and consistency (mean squared difference of models learned) in experiments 1068 and 1116. Training took as input an example matrix **X =** {*x*_*ab*_} where rows {*x*_*a*_}_*a* = 1…*N*_ are example vectors with *b* = 125,509 values. The input matrix **X** is a transformation of fluorescent intensity matrix **X** ′  **=** {*x*′_*ab*_} where the transformation is

$$ \mathbf{X}={\left\{{\log}_{10}\left(\frac{x_a^{\prime }+100}{\mathrm{median}\left({x}_a^{\prime }+100\right)}\right)\ \right\}}_{a=1\dots N}. $$

Additional inputs included label column vector **y**, which was donor’s chronological age and input parameters *λ* and *α*, which act as regularizer and L1-vs-L2 norm weighting, respectively. We then learn weighting vector *β* that minimizes loss function *R*, defined as

$$ R=0.5{\left\Vert \mathbf{y}-\mathbf{X}\beta \right\Vert}_2^2+\lambda\ \left(\ \alpha {\left\Vert \beta \right\Vert}_1+0.5\left(1-\alpha \right){\left\Vert \beta \right\Vert}_2\ \right), $$

with *λ* > 0, *α* ∈ [0, 1], ‖*β*‖_*p*_ is p-norm of *β*. Early cross-validation studies on training sets found that alpha = 0.05 and *λ* ∈ [0.001,10] maximized Pearson’s correlation with chronological age. However, a broad range of parameters *λ*, *α* yielded similar results (Figure S6A). To increase reproducibility, we performed hyperparameter search on reproducibility metrics, leading to higher regularization (lambda > 1) and increased weighting towards L2 norm vs. L1 norm. This tilted error toward models that were “underfit” and “denser”, which resulted in models with lower variance and increased reproducibility (Figure S6A).

### Technical validation and reproducibility of age-associated antibody binding

The multi-serine binding and machine learning model for chronological age prediction were both validated using arrays from independently manufactured wafer batches and reagents. The peptide microarray assay was performed by-hand and by the automated integrated system. Samples were processed in a variety of manner and comparable results were found (see column filtering).

### Interfering substance spike-in experiments

To determine if common serum components known to interfere in immunoassays influenced the immune age measurement, we compared the immune age of samples with and without the addition of six common interferants (Sun Diagnostics, New Gloucester, ME). Prior to the assay, contrived samples for four donors were prepared with the following neat sample concentrations: triglycerides (500.0 mg/dL), rheumatoid factor (RF) (1000.0 IU/ml), conjugated bilirubin (5.0 mg/dL), human anti-mouse antibody (1000.0 ng/ml), hemoglobin (2000.0 mg/dL), and unconjugated bilirubin (15.0 mg/dL). The contrived sample was diluted to a final sample concentration of 1:625 and assayed as described above.

### Molecular size fractionation by centrifugal column filters

To determine the impact of small molecules on IgG binding to peptide arrays, we performed size fractionation of serum samples and re-assayed individual serum fractions. Serum samples were diluted 1:300 in PBST and spun at 5000G for 1 min on Amicon Ultra-0.5 mL 30 K centrifugal filters (MilliporeSigma). While the 30 K filters nominally separate molecules < 30 kDa into the flow-through and retain molecules > 30 kDa in the filtrate, actual concentration/depletion was confirmed with Coomassie Blue staining.

## Supplementary information

**Additional file 1:** **Figure S1.** Age and BMI are associated with antibody binding profiles. (A) Verification Cohort multisite recruitment was concentrated in a subset of states. (B,C) Examples of the many probes with fluorescent intensities (y-axes) that are correlated with age (B) or BMI (C). (D) Volcano plots show that many probes have statistically significant effect size for age and BMI. *P*, *q* (FDR); the Bonferroni estimate of *P*_*FWER*_ cutoffs are shown as dashed lines. FWER is the family-wise error rate.

**Additional file 2:** **Figure S2.** Peptide array fluorescent intensity is highly reproducible across reagent lots, independent cohorts, and assay batch. (A) To estimate reagent lot and assay batch impact, 66 donors from the Discovery Cohort were selected for repeat assay using arrays from an independent manufacturing synthesis batch. The initial quantification (x-axis) is shown for each probe (dot) for all donors (single scatter plot) compared to re-assay on an independent reagent lot (y-axis). Values shown are normalized intra-array by median and inter-array by probe mean. This normalization decreases artifactual correlation as a result of probe absolute quantification, which tends to be similar in many conditions. Thus, each scatter plot shows the donor-specific residuals, which are highly correlated in the initial and repeated quantification. (B,C) Age (B) and BMI (C) statistics are reproducible across multiple independent cohorts. Age is highly reproducible with nearly exact same large effect size for each probe across cohorts. BMI is reproducible, but with smaller overall effect size and increased variation in BMI log10 fold change. Axes show average log10 ratio and each data point is a peptide probe on the array. (D) Probe correlations and association with age are reproducible across reagent lot and assay batch. Donors are the 66 selected for technical replication (described in main text), probes are same as shown in main-text heatmap, axis clustering uses new technical replicate data using independent reagent and array synthesis lots.

**Additional file 3:** **Figure S3.** Peptide probe motifs that are associated with ageing and BMI. (A) Peptide sequences that are associated with age, scatter plot same as in Fig. 1d. (B) Peptide sequences that are associated with BMI. (C) Sequence motifs in peptide probes associated with BMI. Probes associated with BMI typically have N-terminus glutamic acid (N-glutamic acid). Motif information content (bits, y-axis) is shown for each position (x-axis). (D) The N-terminus di-serine and glutamic acid motifs are present in nearly all age- and BMI-associated probes, respectively, whereas these motifs are much less common across non-age and non-BMI associated probes. Fraction of probes (y-axis) with specified motifs (color legend) are shown for age, BMI, and all other probes (x-axis). (E) The presence of N-terminus glutamic acid (E) residues is associated with BMI-correlated probes. A single E residue is comparable in significance to multiple E residues. The presence of glutamic acid at the N-terminus is significantly associated with BMI, whereas any other position in the peptide probe has limited association with BMI. (F) The di-serine score is similar across array formats. Donor samples were assayed on standard sized arrays (131 k probes, x-axis) and large-format arrays (3366 k probes y-axis) to find that the presence of N-terminus di-serine motif conferred similar association with age (axis values). Each data point represents a single donor. (G) On the 3366 k array format with both acetyl capped and uncapped probes (351 k array format, see Methods), age-associated probes are N-terminus acetylated capped. Each data point shows probe count (y-axis) for a single cutoff value to consider a probe to be age associated (x-axis). (H) Donor samples were assayed on arrays with 100% acetylated probes (x-axis) and arrays where only a fraction of probes where acetylated (y-axis). Probes that are acetylated in the split array (red) had higher association with age than those probes uncapped on the split array (black). This intra-array experimental design controls for potential inter-array confounders. Each data point represents a single probe and axes are average values across donors of specified age groups.

**Additional file 4:** **Figure S4.** Illustration of how age and BMI can be used as proxies for immunosenescence, where regression residuals are values of interest. Hypothetical data are shown, emphasizing residuals with respect to a learned regression line. Dots are hypothetical donors (this is not real data) that have been labeled with an Immune Wellness proxy (x-axis, proxies selected are age and BMI) from which an immune age (y-axis) was learned. Three hypothetical donors are highlighted and their signed residuals are plotted on the right with interpretations of good, average, and poor immune wellness.

**Additional file 5:** **Figure S5.** The regression model and residuals are consistent across multiple training and verification cohorts and potential confounding variables (array synthesis, ethnicity, sample collection site, and BMI). (A) The age regression residuals are consistent across multiple sub-training sets. The Training Cohort and Verification Cohorts were merged into a single large cohort (*N* = 1675). Two training sets were created, each of size 698, which left 279 samples as a holdout set. An elastic net regression model was trained on each of the training sets and then residuals were calculated on the holdout set of 279 samples (data points) using each of the models (axes). High correlation between model residuals suggests a low variance-error term, which is consistent with residuals potentially being biologically relevant. Result is representative of 100 simulated training set splits. (B) Age regression residuals (axes) are correlated when samples are assayed on different array formats, different training sets, and different algorithm parameters (subplots). Each dot is a single donor assayed for a single permutation of array type, training set, and algorithm parameters. Not all samples were assayed on all permutations of array type. Values on x- and y-axes are residuals, which normalize out the default transitive correlation of all models being correlated to chronological age. (C) Regression model yields similar results across samples binned by ethnicity and sample collection site. Each dot is single donor and each line shows regression predictions on grouped donors. Chronological age (x-axis) and prediction of age based on peptide array regression model (y-axis) are shown. Legend shows correlation coefficient, regression slope and intercept, and number of samples in a given bin. Data shown is regression model learned on Training Cohort and applied to the Verification Cohort. (D) The intercept (shift) and slope (interacting) terms associated with BMI, ethnicity, and collection state are not statistically significant. One-way ANOVA test statistics are shown in the Table. (E) Age-associated antibody-peptide binding to di-serine N-terminus peptides is highly consistent in a long-term study. A total of *N* = 16 donors consented to regular blood draws for > 1 yr. Donors with > 5 samples over > 1 yr (*N* = 13) had consistent age-regression values over this time period. Data shown for all donors (lines).

**Additional file 6:** **Figure S6.** Parameter selection and impact on accuracy and analytic characteristics of the regression model. (A) Regression accuracy and stability metrics (rows) are impacted by parameters alpha (columns) and lambda (x-axis). While accuracy (quantified by Pearson’s correlation r) is similar across many parameters, residual correlation and MSE can vary substantially. We select α = 0.01 and λ = 1 based on this analysis. Note that the residual correlation plot is undefined (currently shown as y = 1) when number of features in model is zero. Empty plots showing “number of features” on y-axis indicate that even for high λ = 10, we found > 1500 features included in model. (B) As training set size increases (x-axis) the learned model has improved accuracy as measured by Pearson’s correlation with chronological age (y-axis). Each training set is simulated 10 times from all 1675 samples using a holdout set to test for correlation with chronological age.

**Additional file 7:** **Figure S7.** Peptide array immune age is distinct from cytokine derived immune age. (A,B) Correlation between cytokine marker quantification by Luminex assay (y-axis, log-scale) and age (A) and BMI (B). (C) Peptide array prediction of age is not improved by cytokine data; however, prediction of BMI is significantly increased when including cytokine data. Graphs show on the y-axis, the relationship between peptide array signals (row a), cytokines (row b), and combinations of peptide array and cytokines (rows c and d) and on the x-axis, a combination function of chronological age and BMI that roughly approximates health (column i), chronological age (column ii) and BMI (column iii), as proxies for immune health. Row (c) trained on example matrix where peptide array and cytokine data were concatenated, whereas row (d) trained on matrix where only score derived from peptide array data was concatenated to cytokine data. Cytokine data was transformed by log10(x + 1) to make linear regression variance more homoscedastic. In this context, “concatenation” refers to combining two matrices (organized as donors as rows and measurements in columns) by adjoining column-wise after matching rows by donor. (D) Chronological age prediction by peptide array (y-axis) and cytokine levels (x-axis) finds that markers of humoral and innate immunity have related, but independent prediction of chronological age.

**Additional file 8:** **Figure S8.** Additional control experiments suggest that age-associated antibody-peptide binding is driven by direct IgG binding. (A) Original image associated with Fig. 4b. In addition to filter columns, we also demonstrated IgG separation with Melon Gel purification; however, the Melon Gel purification assay reagents disrupted IgG-peptide array binding in the recombined fractions (the positive control) even though it produced superior IgG purification. It was thus excluded from downstream analysis. (B) The age-associated probes are not bound by anti-IgG secondary antibody, which is used for detecting IgG bound to peptides. Generic stickiness or peptides with similarity to IgG-Fc (which can partially bind secondary-antibody directly; y-axis) have minimal correlation with on age-association (x-axis). Each data point is a probe. (C-D) Interfering substances found in varying abundances in serum have limited impact on age-associated peptide probes. Serum from 4 healthy donors with and without an interfering substance was assayed by peptide microarray. Triglycerides, rheumatoid factor (RF), conjugated bilirubin, human anti-mouse antibody (HAMA), hemoglobin, and unconjugated bilirubin at a single high concentration (Methods). (C) The log10 ratio of serum with and without interfering substance (y-axis) is compared to the log10 ratio of serum from older (> 60 years) and younger (< 40 years) donors (x-axis). The only interfering substance that shows similar impact on peptides is RF, which is not statistically significant (*p* < 0.06, enrichment ratio of 1.9) as calculated by Fisher’s exact test (cutoffs were age log10 ratio > 0.25 and RF log10 ratio > 0.05; other cutoffs provided similar values). Pearson’s and Spearman correlation were not near significance, *r* = 0.06, rho = 0.03 (*p* < 0.54 and *p* < 0.69, respectively). (D) Similar to (C), except x-axis now shows the log10 ratio of serum from higher BMI (> 30) vs lower BMI (< 25). No interferants achieved statistical significance (using cutoff of *p* < 0.05).

**Additional file 9:** **Table S1.** The complete dataset for the Training Cohort (Experiment 1068). Dataset consists of meta data, raw foreground intensity data, and transformed data on which most analyses were performed. Meta data (META) uses row name ‘scan id’ as unique identifier that can be matched with other data files. Meta data columns include peptide array reagent, batch, assay, infection, and demographic information for a given sample. The foreground (FG) intensity values are formatted with rows as peptide probes and columns as donors. Each value is in range of [0,65,535]. The first column has row names, which is probe name, underscore, peptide sequence, where probe name is created based on the “X” and “Y” physical locations of where the probe is found on the array. The first row has column name ‘scan id’, which can be matched to the META rownames. The transformed data (LFG) is log-transformed data with shrinkage: log10(x + 100). This transform helps data follow a more homoskedastic distribution and decreases the influence of low intensity probes.

**Additional file 10:** **Table S2.** Same as Table S1, but for Technical Reproducibility Cohort (Experiment 1102).

**Additional file 11:** **Table S3.** Same as Table S1, but for Verification Cohort (Experiment 1116).

**Additional file 12:** **Table S4.** Same as Table S1, but for joint cohort rerun on improved array manufacturing process (Experiment 1375).

**Additional file 13:** **Table S5.** Association statistics for age and BMI with peptide probe intensity for the Training Cohort (Experiment 1068).

**Additional file 14:** **Table S6.** Mapping from self-reported ethnicities to grouped ethnicities.

**Additional file 15:** **Appendix** [49–52].

## Data Availability

The datasets generated and/or analysed during the current study are available in the FigShare repository, 10.6084/m9.figshare.c.5111282.v1.

## Abbreviations

131 K: The 131,000 feature peptide array
351 K: The 351,909 feature peptide array
3366 K: The 3,366,522 feature peptide array
ANOVA: Analysis of Variance
BMI: Body Mass Index
CTS: Creative Testing Solutions
dsDNA: Double stranded DNA
h: Hour
kDa: Kilodalton
min: Minute
mm: Millimeter
mL: Milliliter
dL: Deciliter
μL: Microliter
PBST+P: Phosphate Buffered Saline, 0.05% Tween 20, 0.1% Proclin 950
RA: Rheumatoid Arthritis
RF: Rheumatoid Factor
SLE: Systemic Lupus Erythematosus
SLEDAI: Systemic Lupus Erythematosus Disease Activity
SS: Di-serine

## References

[1] Gavazzi G, Krause KH (2002). Ageing and infection. Lancet Infect Dis.

[2] Gross PA, Hermogenes AW, Sacks HS, Lau J, Levandowski RA (1995). The efficacy of influenza vaccine in elderly persons. A meta-analysis and review of the literature. Ann Intern Med.

[3] Siegrist CA, Aspinall R (2009). B-cell responses to vaccination at the extremes of age. Nat Rev Immunol.

[4] Goronzy JJ, Weyand CM (2017). Successful and maladaptive T cell aging. Immunity.

[5] Frasca D, Blomberg BB (2009). Effects of aging on B cell function. Curr Opin Immunol.

[6] Linton PJ, Haynes L, Klinman NR, Swain SL (1996). Antigen-independent changes in naive CD4 T cells with aging. J Exp Med.

[7] Weiskopf D, Weinberger B, Grubeck-Loebenstein B (2009). The aging of the immune system. Transpl Int.

[8] Naylor K, Li G, Vallejo AN, Lee WW, Koetz K, Bryl E, Witkowski J, Fulbright J, Weyand CM, Goronzy JJ (2005). The influence of age on T cell generation and TCR diversity. J Immunol.

[9] Hu A, Ehleiter D, Ben-Yehuda A, Schwab R, Russo C, Szabo P, Weksler ME (1993). Effect of age on the expressed B cell repertoire: role of B cell subsets. Int Immunol.

[10] Plowden J, Renshaw-Hoelscher M, Engleman C, Katz J, Sambhara S (2004). Innate immunity in aging: impact on macrophage function. Aging Cell.

[11] Nikolich-Žugich J, Li G, Uhrlaub JL, Renkema KR, Smithey MJ (2012). Age-related changes in CD8 T cell homeostasis and immunity to infection. Semin Immunol.

[12] Hearps AC, Martin GE, Angelovich TA, Cheng WJ, Maisa A, Landay AL, Jaworowski A, Crowe SM (2012). Aging is associated with chronic innate immune activation and dysregulation of monocyte phenotype and function. Aging Cell.

[13] Gibson KL, Wu YC, Barnett Y, Duggan O, Vaughan R, Kondeatis E, Nilsson BO, Wikby A, Kipling D, Dunn-Walters DK (2009). B-cell diversity decreases in old age and is correlated with poor health status. Aging Cell.

[14] Dunn-Walters DK, O’Hare JS, Fulop T, Franceschi C, Hirokawa K, Pawelec G (2019). Older Human B Cells and Antibodies. Handbook of Immunosenescence: Basic Understanding and Clinical Implications.

[15] Frasca D, Blomberg BB (2011). Aging affects human B cell responses. J Clin Immunol.

[16] Goenka R, Scholz JL, Naradikian MS, Cancro MP (2014). Memory B cells form in aged mice despite impaired affinity maturation and germinal center kinetics. Exp Gerontol.

[17] Britanova OV, Putintseva EV, Shugay M, Merzlyak EM, Turchaninova MA, Staroverov DB, Bolotin DA, Lukyanov S, Bogdanova EA, Mamedov IZ (2014). Age-related decrease in TCR repertoire diversity measured with deep and normalized sequence profiling. J Immunol.

[18] Palmer S, Albergante L, Blackburn CC, Newman TJ (2018). Thymic involution and rising disease incidence with age. Proc Natl Acad Sci U S A.

[19] Vaziri H, Dragowska W, Allsopp RC, Thomas TE, Harley CB, Lansdorp PM (1994). Evidence for a mitotic clock in human hematopoietic stem cells: loss of telomeric DNA with age. Proc Natl Acad Sci U S A.

[20] Pang WW, Price EA, Sahoo D, Beerman I, Maloney WJ, Rossi DJ, Schrier SL, Weissman IL (2011). Human bone marrow hematopoietic stem cells are increased in frequency and myeloid-biased with age. Proc Natl Acad Sci U S A.

[21] Geiger H, de Haan G, Florian MC (2013). The ageing haematopoietic stem cell compartment. Nat Rev Immunol.

[22] Herrero C, Sebastián C, Marqués L, Comalada M, Xaus J, Valledor AF, Lloberas J, Celada A (2002). Immunosenescence of macrophages: reduced MHC class II gene expression. Exp Gerontol.

[23] Linton PJ, Thoman ML (2014). Immunosenescence in monocytes, macrophages, and dendritic cells: lessons learned from the lung and heart. Immunol Lett.

[24] Shaw AC, Goldstein DR, Montgomery RR (2013). Age-dependent dysregulation of innate immunity. Nat Rev Immunol.

[25] Howard WA, Gibson KL, Dunn-Walters DK (2006). Antibody quality in old age. Rejuvenation Res.

[26] Dunn-Walters DK, Banerjee M, Mehr R (2003). Effects of age on antibody affinity maturation. Biochem Soc Trans.

[27] Nicoletti C, Yang X, Cerny J (1993). Repertoire diversity of antibody response to bacterial antigens in aged mice. III. Phosphorylcholine antibody from young and aged mice differ in structure and protective activity against infection with Streptococcus pneumoniae. J Immunol.

[28] Bovbjerg DH, Kim YT, Schwab R, Schmitt K, DeBlasio T (1991). Weksler ME: "cross-wiring" of the immune response in old mice: increased autoantibody response despite reduced antibody response to nominal antigen. Cell Immunol.

[29] Zhao KS, Wang YF, Guéret R, Weksler ME (1995). Dysregulation of the humoral immune response in old mice. Int Immunol.

[30] Weksler ME (2000). Changes in the B-cell repertoire with age. Vaccine.

[31] Rowley MJ, Buchanan H, Mackay IR (1968). Reciprocal change with age in antibody to extrinsic and intrinsic antigens. Lancet.

[32] Rowe M, Melnick J, Gerwien R, Legutki JB, Pfeilsticker J, Tarasow TM, Sykes KF (2017). An ImmunoSignature test distinguishes Trypanosoma cruzi, hepatitis B, hepatitis C and West Nile virus seropositivity among asymptomatic blood donors. PLoS Negl Trop Dis.

[33] NLM format citation: Kuo CL, Pilling LC, Kuchel GA, Ferrucci L, Melzer D. Telomere length and aging-related outcomes in humans: A Mendelian randomization study in 261,000 older participants. Aging Cell. 2019;18(6):e13017. 10.1111/acel.13017. Epub 2019 Aug 24. PMID: 31444995; PMCID: PMC6826144. 10.1111/acel.13017 PMC6826144 31444995

[34] Ganna A, Ingelsson E (2015). 5 year mortality predictors in 498,103 UK biobank participants: a prospective population-based study. Lancet.

[35] Needham BL, Rehkopf D, Adler N, Gregorich S, Lin J, Blackburn EH, Epel ES (2015). Leukocyte telomere length and mortality in the National Health and nutrition examination survey, 1999-2002. Epidemiology.

[36] Chen BH, Marioni RE, Colicino E, Peters MJ, Ward-Caviness CK, Tsai PC, Roetker NS, Just AC, Demerath EW, Guan W (2016). DNA methylation-based measures of biological age: meta-analysis predicting time to death. Aging (Albany NY).

[37] Marioni RE, Shah S, McRae AF, Chen BH, Colicino E, Harris SE, Gibson J, Henders AK, Redmond P, Cox SR (2015). DNA methylation age of blood predicts all-cause mortality in later life. Genome Biol.

[38] Varadhan R, Yao W, Matteini A, Beamer BA, Xue QL, Yang H, Manwani B, Reiner A, Jenny N, Parekh N (2014). Simple biologically informed inflammatory index of two serum cytokines predicts 10 year all-cause mortality in older adults. J Gerontol A Biol Sci Med Sci.

[39] Ridker PM, Everett BM, Thuren T, MacFadyen JG, Chang WH, Ballantyne C, Fonseca F, Nicolau J, Koenig W, Anker SD (2017). Antiinflammatory therapy with Canakinumab for atherosclerotic disease. N Engl J Med.

[40] Alpert A, Pickman Y, Leipold M, Rosenberg-Hasson Y, Ji X, Gaujoux R, Rabani H, Starosvetsky E, Kveler K, Schaffert S (2019). A clinically meaningful metric of immune age derived from high-dimensional longitudinal monitoring. Nat Med.

[41] Earls JC, Rappaport N, Heath L, Wilmanski T, Magis AT, Schork NJ, Omenn GS, Lovejoy J, Hood L, Price ND (2019). Multi-Omic Biological Age Estimation and Its Correlation With Wellness and Disease Phenotypes: A Longitudinal Study of 3,558 Individuals. J Gerontol A Biol Sci Med Sci.

[42] Zou H, Hastie T (2005). Regularization and variable selection via the elastic net. J R Stat Soc Ser B (Statistical Methodology).

[43] Corper AL, Sohi MK, Bonagura VR, Steinitz M, Jefferis R, Feinstein A, Beale D, Taussig MJ, Sutton BJ (1997). Structure of human IgM rheumatoid factor fab bound to its autoantigen IgG fc reveals a novel topology of antibody-antigen interaction. Nat Struct Biol.

[44] Duquerroy S, Stura EA, Bressanelli S, Fabiane SM, Vaney MC, Beale D, Hamon M, Casali P, Rey FA, Sutton BJ (2007). Crystal structure of a human autoimmune complex between IgM rheumatoid factor RF61 and IgG1 fc reveals a novel epitope and evidence for affinity maturation. J Mol Biol.

[45] NLM format citation: Fahy GM, Brooke RT, Watson JP, Good Z, Vasanawala SS, Maecker H, Leipold MD, Lin DTS, Kobor MS, Horvath S. Reversal of epigenetic aging and immunosenescent trends in humans. Aging Cell. 2019;18(6):e13028. 10.1111/acel.13028. Epub 2019 Sep 8. PMID: 31496122; PMCID: PMC6826138. 10.1111/acel.13028 PMC6826138 31496122

[46] Weyand CM, Goronzy JJ (2016). Aging of the Immune System. Mechanisms and Therapeutic Targets. Ann Am Thorac Soc.

[47] Montecino-Rodriguez E, Berent-Maoz B, Dorshkind K (2013). Causes, consequences, and reversal of immune system aging. J Clin Invest.

[48] Marko MG, Ahmed T, Bunnell SC, Wu D, Chung H, Huber BT, Meydani SN (2007). Age-associated decline in effective immune synapse formation of CD4(+) T cells is reversed by vitamin E supplementation. J Immunol.

[49] Bonagura VR, Agostino N, Børretzen M, Thompson KM, Natvig JB, Morrison SL (1998). Mapping IgG epitopes bound by rheumatoid factors from immunized controls identifies disease-specific rheumatoid factors produced by patients with rheumatoid arthritis. J Immunol.

[50] Van Esch WJ, Reparon-Schuijt CC, Hamstra HJ, Van Kooten C, Logtenberg T, Breedveld FC, Verweij CL (2003). Human IgG fc-binding phage antibodies constructed from synovial fluid CD38+ B cells of patients with rheumatoid arthritis show the imprints of an antigen-dependent process of somatic hypermutation and clonal selection. Clin Exp Immunol.

[51] Prokunina L, Padyukov L, Bennet A, de Faire U, Wiman B, Prince J, Alfredsson L, Klareskog L, Alarcón-Riquelme M (2004). Association of the PD-1.3A allele of the PDCD1 gene in patients with rheumatoid arthritis negative for rheumatoid factor and the shared epitope. Arthritis Rheum.

[52] Westwood OM, Nelson PN, Hay FC (2006). Rheumatoid factors: what's new?. Rheumatology (Oxford).

