# Supplementary figures and images for: Age-associated changes in the circulating human antibody repertoire are upregulated in autoimmunity
Source: Immun Ageing. 2020 Oct 6;17:28. doi: 10.1186/s12979-020-00193-x (PMC7539520; doi:10.1186/s12979-020-00193-x)

Figure 1 - Supp 1

A

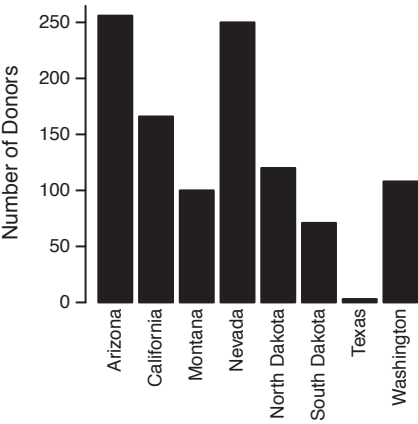

B

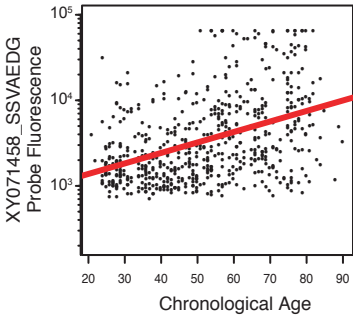

C

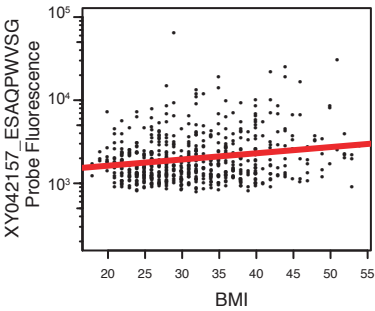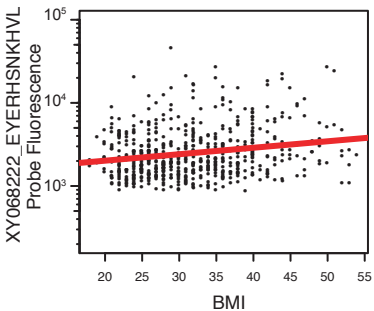

D

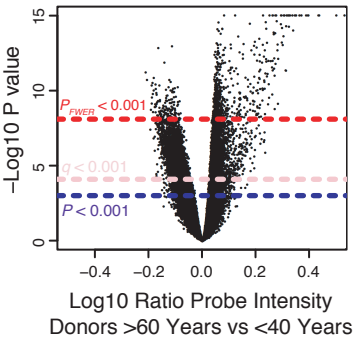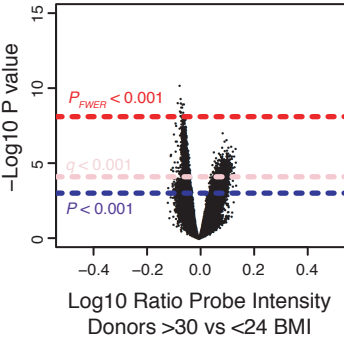

Supplement: Supplementary file 1 — Additional file 1: Figure S1. Age and BMI are associated with antibody binding profiles. (A) Verification Cohort multisite recruitment was concentrated in a subset of states. (B,C) Examples of the many probes with fluorescent intensities (y-axes) that are correlated with age (B) or BMI (C). (D) Volcano plots show that many probes have statistically significant effect size for age and BMI. P, q (FDR); the Bonferroni estimate of PFWER cutoffs are shown as dashed lines. FWER is the family-wise error rate. [file 12979_2020_193_MOESM1_ESM.pdf]

Figure 1 - Supp 2

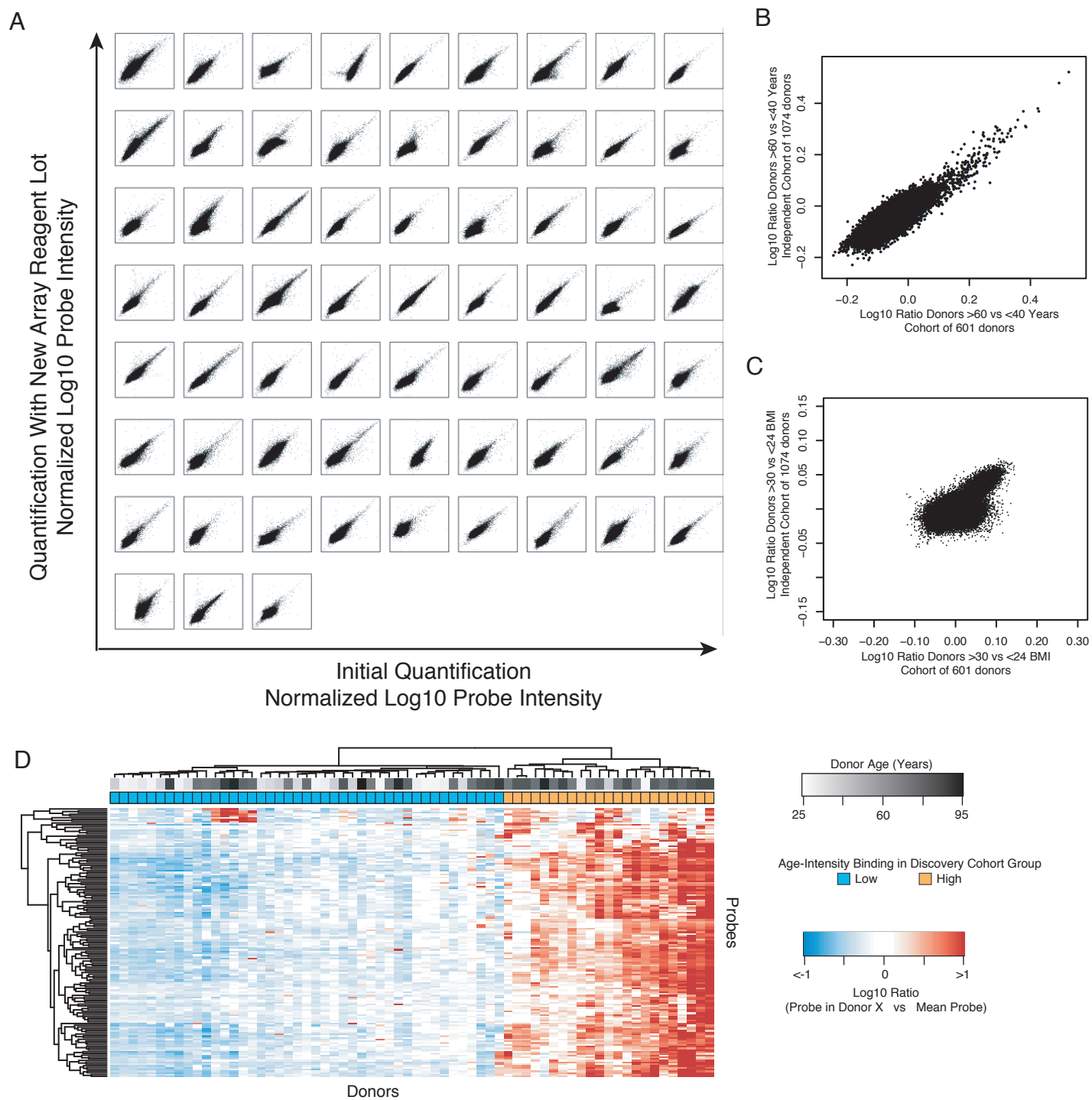

Supplement: Supplementary file 2 — Additional file 2: Figure S2. Peptide array fluorescent intensity is highly reproducible across reagent lots, independent cohorts, and assay batch. (A) To estimate reagent lot and assay batch impact, 66 donors from the Discovery Cohort were selected for repeat assay using arrays from an independent manufacturing synthesis batch. The initial quantification (x-axis) is shown for each probe (dot) for all donors (single scatter plot) compared to re-assay on an independent reagent lot (y-axis). Values shown are normalized intra-array by median and inter-array by probe mean. This normalization decreases artifactual correlation as a result of probe absolute quantification, which tends to be similar in many conditions. Thus, each scatter plot shows the donor-specific residuals, which are highly correlated in the initial and repeated quantification. (B,C) Age (B) and BMI (C) statistics are reproducible across multiple independent cohorts. Age is highly reproducible with nearly exact same large effect size for each probe across cohorts. BMI is reproducible, but with smaller overall effect size and increased variation in BMI log10 fold change. Axes show average log10 ratio and each data point is a peptide probe on the array. (D) Probe correlations and association with age are reproducible across reagent lot and assay batch. Donors are the 66 selected for technical replication (described in main text), probes are same as shown in main-text heatmap, axis clustering uses new technical replicate data using independent reagent and array synthesis lots. [file 12979_2020_193_MOESM2_ESM.pdf]

Figure 2 - Supp 1

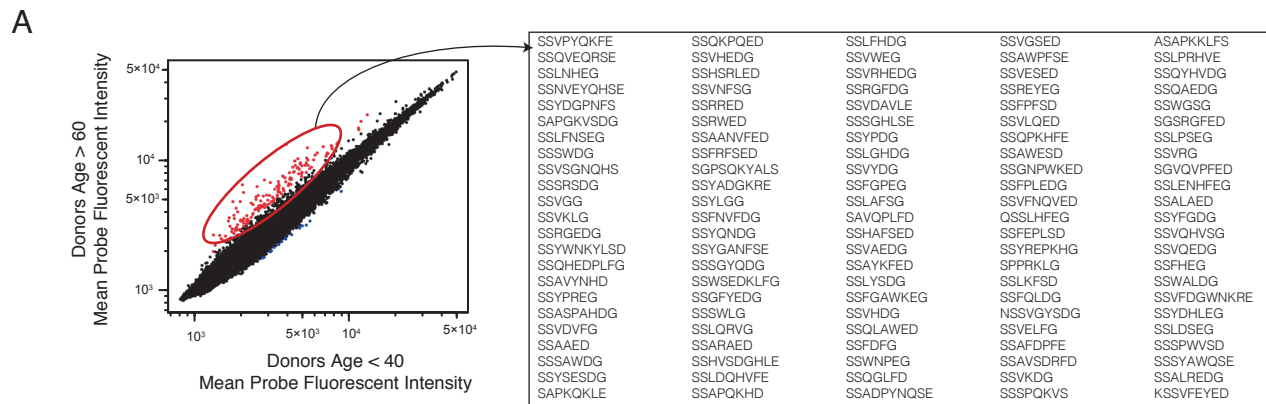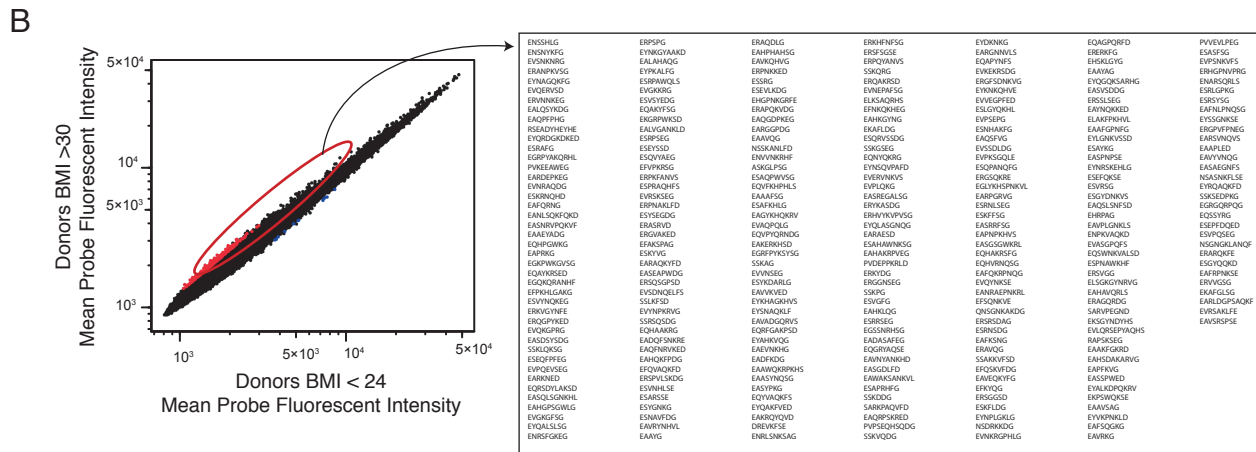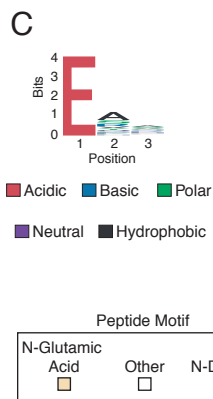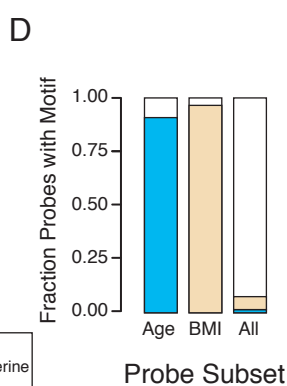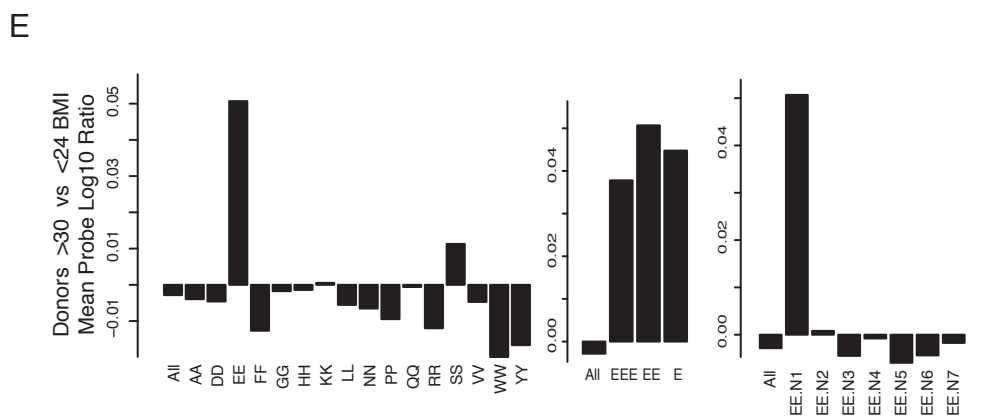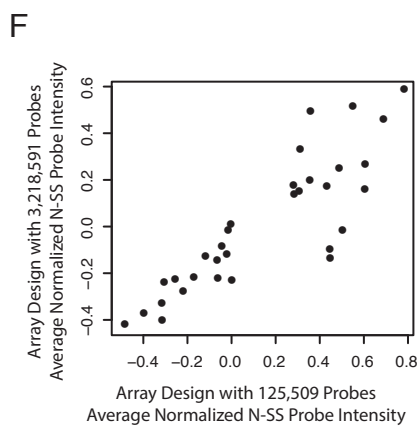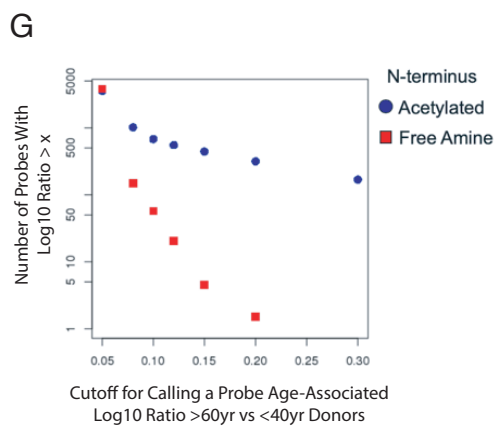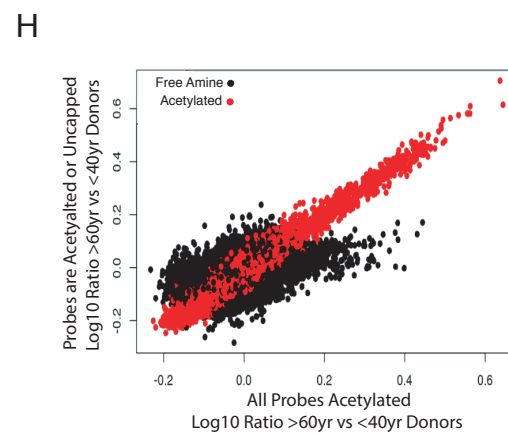

Supplement: Supplementary file 3 — Additional file 3: Figure S3. Peptide probe motifs that are associated with ageing and BMI. (A) Peptide sequences that are associated with age, scatter plot same as in Fig. 1d. (B) Peptide sequences that are associated with BMI. (C) Sequence motifs in peptide probes associated with BMI. Probes associated with BMI typically have N-terminus glutamic acid (N-glutamic acid). Motif information content (bits, y-axis) is shown for each position (x-axis). (D) The N-terminus di-serine and glutamic acid motifs are present in nearly all age- and BMI-associated probes, respectively, whereas these motifs are much less common across non-age and non-BMI associated probes. Fraction of probes (y-axis) with specified motifs (color legend) are shown for age, BMI, and all other probes (x-axis). (E) The presence of N-terminus glutamic acid (E) residues is associated with BMI-correlated probes. A single E residue is comparable in significance to multiple E residues. The presence of glutamic acid at the N-terminus is significantly associated with BMI, whereas any other position in the peptide probe has limited association with BMI. (F) The di-serine score is similar across array formats. Donor samples were assayed on standard sized arrays (131 k probes, x-axis) and large-format arrays (3366 k probes y-axis) to find that the presence of N-terminus di-serine motif conferred similar association with age (axis values). Each data point represents a single donor. (G) On the 3366 k array format with both acetyl capped and uncapped probes (351 k array format, see Methods), age-associated probes are N-terminus acetylated capped. Each data point shows probe count (y-axis) for a single cutoff value to consider a probe to be age associated (x-axis). (H) Donor samples were assayed on arrays with 100% acetylated probes (x-axis) and arrays where only a fraction of probes where acetylated (y-axis). Probes that are acetylated in the split array (red) had higher association with age than those probes uncap [file 12979_2020_193_MOESM3_ESM.pdf]

Figure 3 - Supp 1

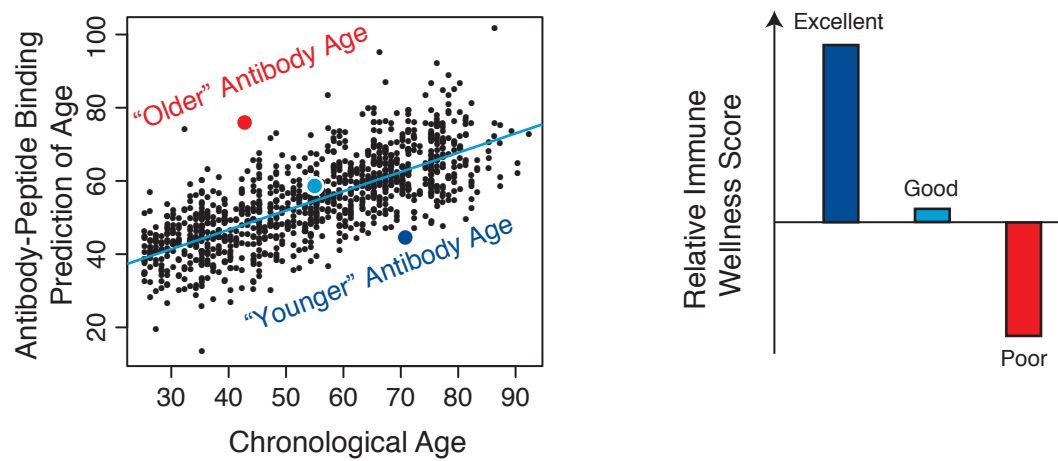

Supplement: Supplementary file 4 — Additional file 4: Figure S4. Illustration of how age and BMI can be used as proxies for immunosenescence, where regression residuals are values of interest. Hypothetical data are shown, emphasizing residuals with respect to a learned regression line. Dots are hypothetical donors (this is not real data) that have been labeled with an Immune Wellness proxy (x-axis, proxies selected are age and BMI) from which an immune age (y-axis) was learned. Three hypothetical donors are highlighted and their signed residuals are plotted on the right with interpretations of good, average, and poor immune wellness. [file 12979_2020_193_MOESM4_ESM.pdf]

Figure 3 - Supp 2

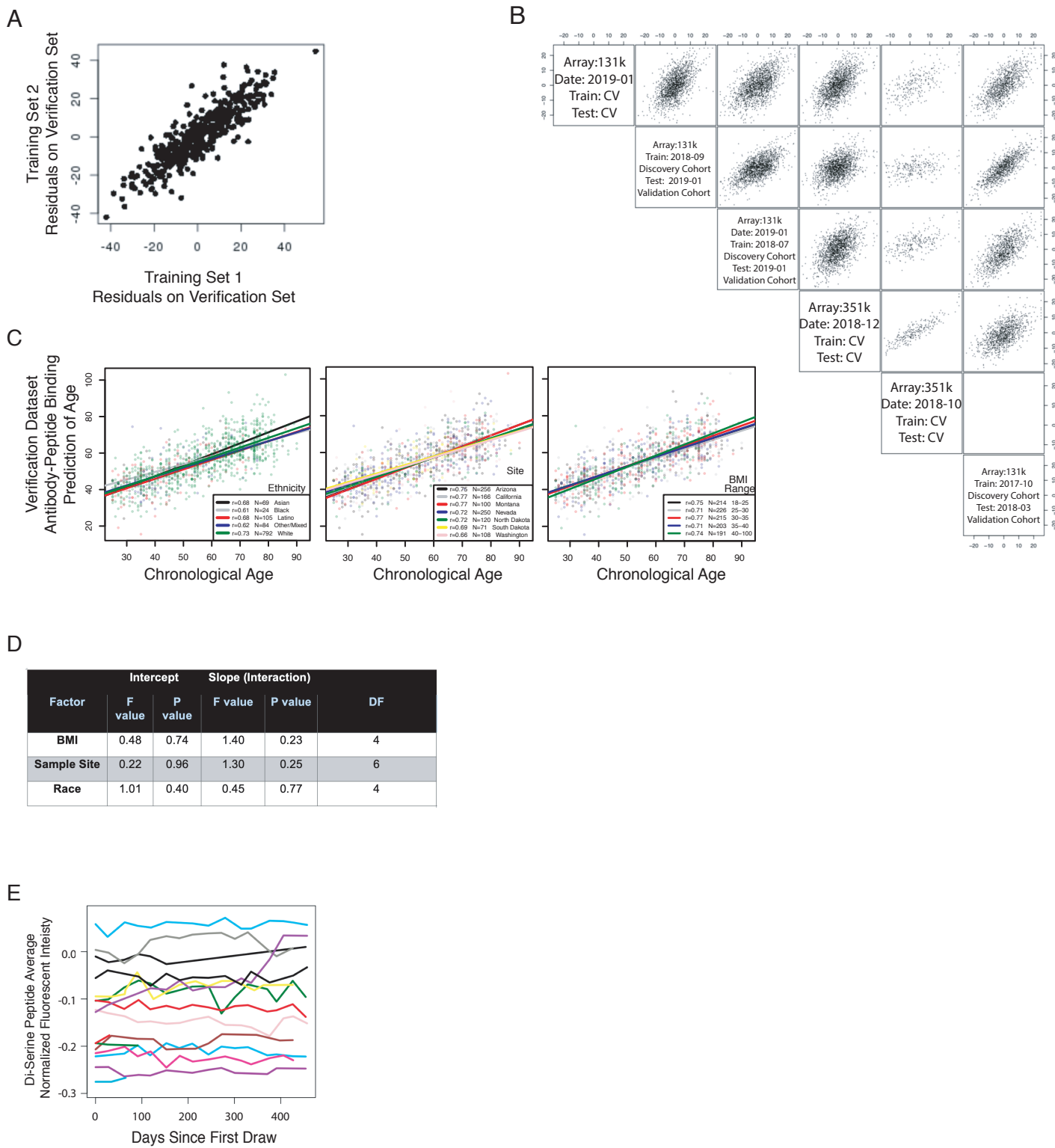

Supplement: Supplementary file 5 — Additional file 5: Figure S5. The regression model and residuals are consistent across multiple training and verification cohorts and potential confounding variables (array synthesis, ethnicity, sample collection site, and BMI). (A) The age regression residuals are consistent across multiple sub-training sets. The Training Cohort and Verification Cohorts were merged into a single large cohort (N = 1675). Two training sets were created, each of size 698, which left 279 samples as a holdout set. An elastic net regression model was trained on each of the training sets and then residuals were calculated on the holdout set of 279 samples (data points) using each of the models (axes). High correlation between model residuals suggests a low variance-error term, which is consistent with residuals potentially being biologically relevant. Result is representative of 100 simulated training set splits. (B) Age regression residuals (axes) are correlated when samples are assayed on different array formats, different training sets, and different algorithm parameters (subplots). Each dot is a single donor assayed for a single permutation of array type, training set, and algorithm parameters. Not all samples were assayed on all permutations of array type. Values on x- and y-axes are residuals, which normalize out the default transitive correlation of all models being correlated to chronological age. (C) Regression model yields similar results across samples binned by ethnicity and sample collection site. Each dot is single donor and each line shows regression predictions on grouped donors. Chronological age (x-axis) and prediction of age based on peptide array regression model (y-axis) are shown. Legend shows correlation coefficient, regression slope and intercept, and number of samples in a given bin. Data shown is regression model learned on Training Cohort and applied to the Verification Cohort. (D) The intercept (shift) and slope (interacting) terms associated with BMI, ethnicit [file 12979_2020_193_MOESM5_ESM.pdf]

Figure 3 - Supp 3

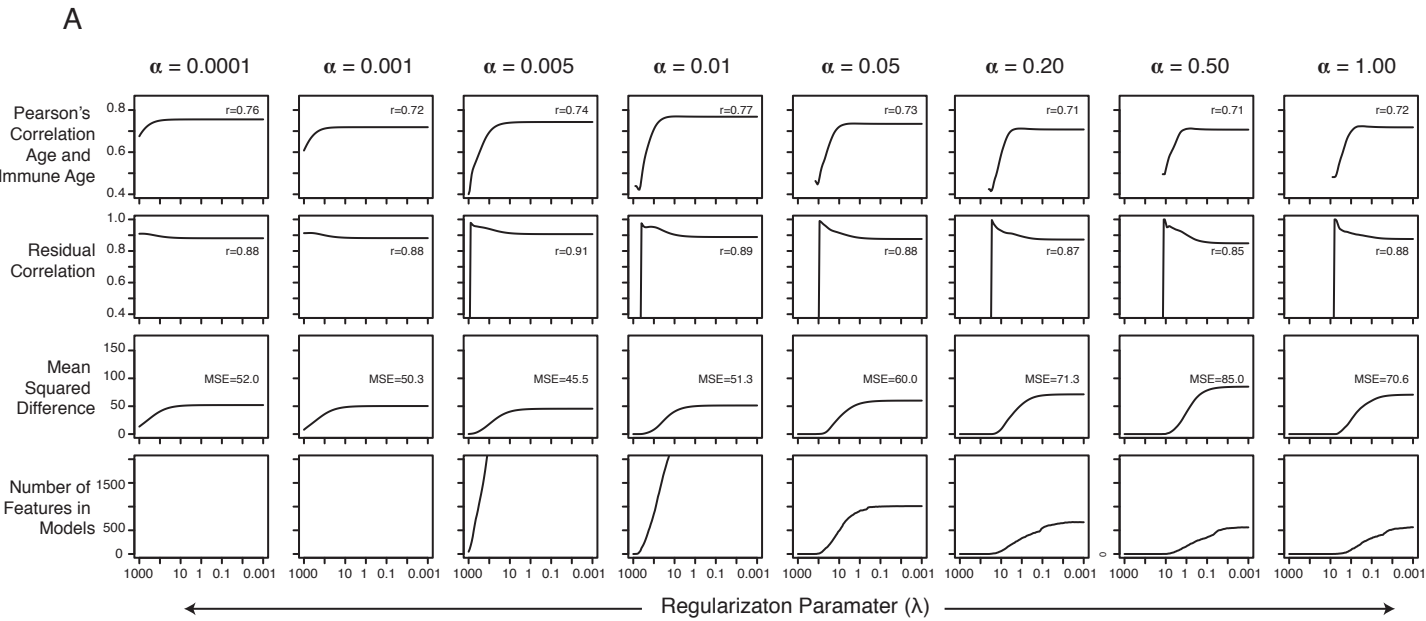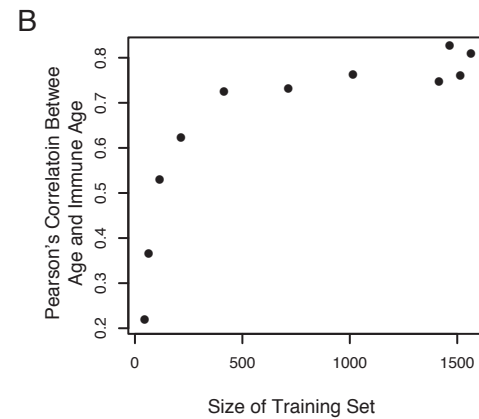

Supplement: Supplementary file 6 — Additional file 6: Figure S6. Parameter selection and impact on accuracy and analytic characteristics of the regression model. (A) Regression accuracy and stability metrics (rows) are impacted by parameters alpha (columns) and lambda (x-axis). While accuracy (quantified by Pearson’s correlation r) is similar across many parameters, residual correlation and MSE can vary substantially. We select α = 0.01 and λ = 1 based on this analysis. Note that the residual correlation plot is undefined (currently shown as y = 1) when number of features in model is zero. Empty plots showing “number of features” on y-axis indicate that even for high λ = 10, we found > 1500 features included in model. (B) As training set size increases (x-axis) the learned model has improved accuracy as measured by Pearson’s correlation with chronological age (y-axis). Each training set is simulated 10 times from all 1675 samples using a holdout set to test for correlation with chronological age. [file 12979_2020_193_MOESM6_ESM.pdf]

Figure 3 - Supp4

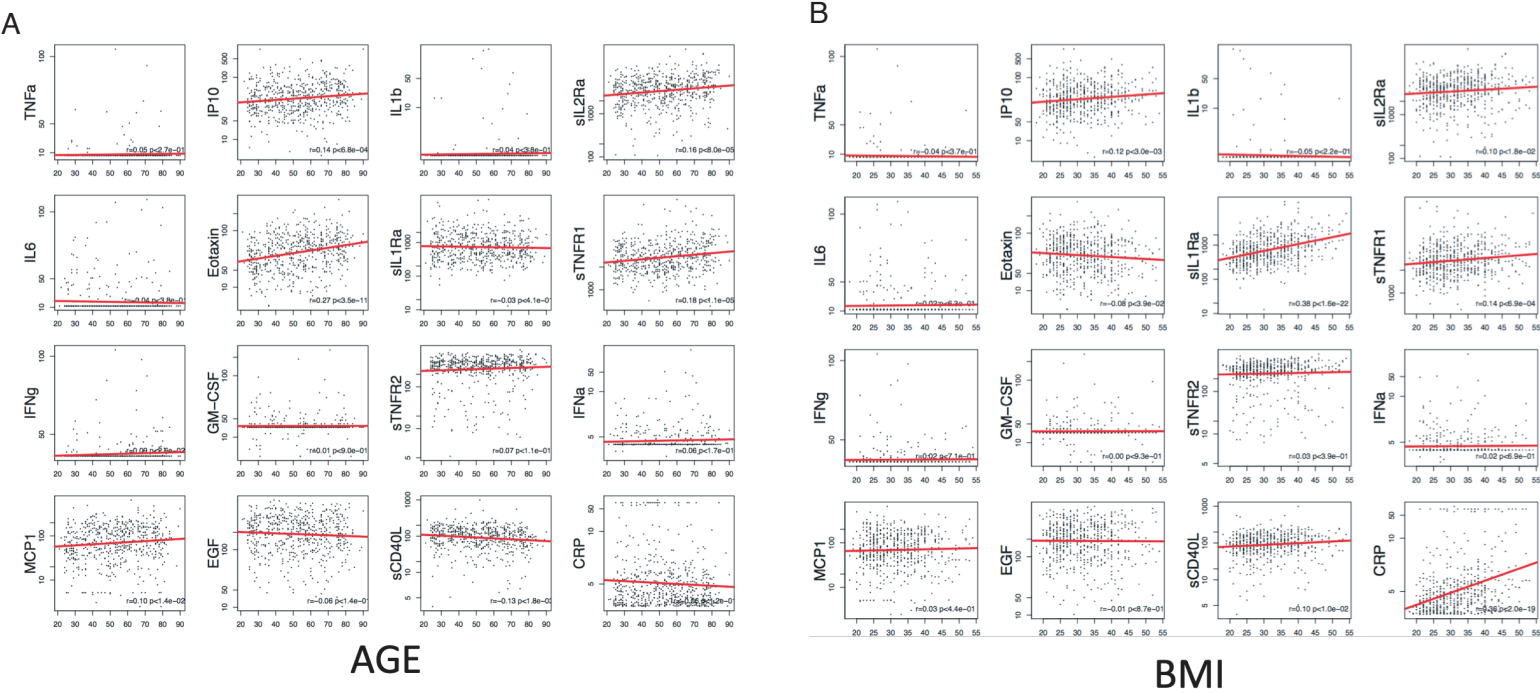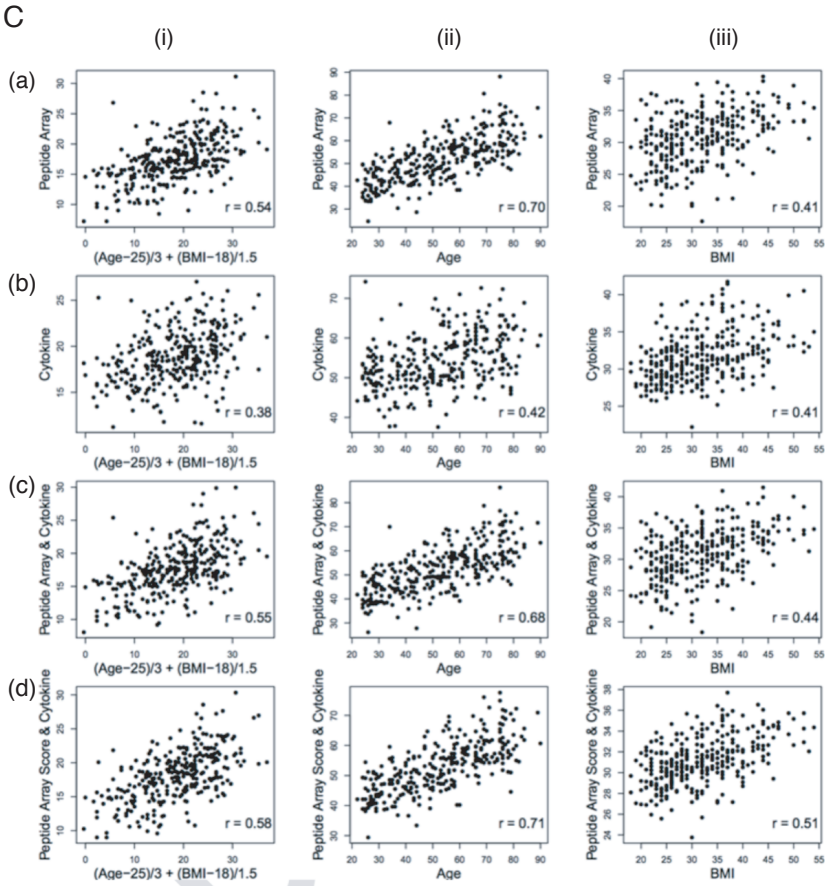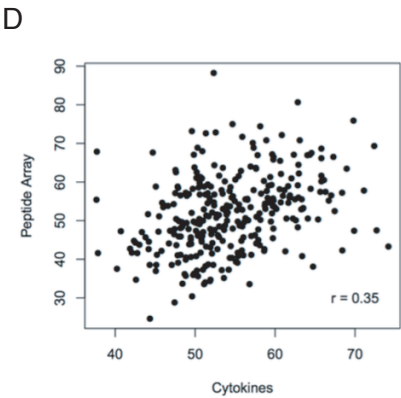

Supplement: Supplementary file 7 — Additional file 7: Figure S7. Peptide array immune age is distinct from cytokine derived immune age. (A,B) Correlation between cytokine marker quantification by Luminex assay (y-axis, log-scale) and age (A) and BMI (B). (C) Peptide array prediction of age is not improved by cytokine data; however, prediction of BMI is significantly increased when including cytokine data. Graphs show on the y-axis, the relationship between peptide array signals (row a), cytokines (row b), and combinations of peptide array and cytokines (rows c and d) and on the x-axis, a combination function of chronological age and BMI that roughly approximates health (column i), chronological age (column ii) and BMI (column iii), as proxies for immune health. Row (c) trained on example matrix where peptide array and cytokine data were concatenated, whereas row (d) trained on matrix where only score derived from peptide array data was concatenated to cytokine data. Cytokine data was transformed by log10(x + 1) to make linear regression variance more homoscedastic. In this context, “concatenation” refers to combining two matrices (organized as donors as rows and measurements in columns) by adjoining column-wise after matching rows by donor. (D) Chronological age prediction by peptide array (y-axis) and cytokine levels (x-axis) finds that markers of humoral and innate immunity have related, but independent prediction of chronological age. [file 12979_2020_193_MOESM7_ESM.pdf]

Figure 4 - Supp 1

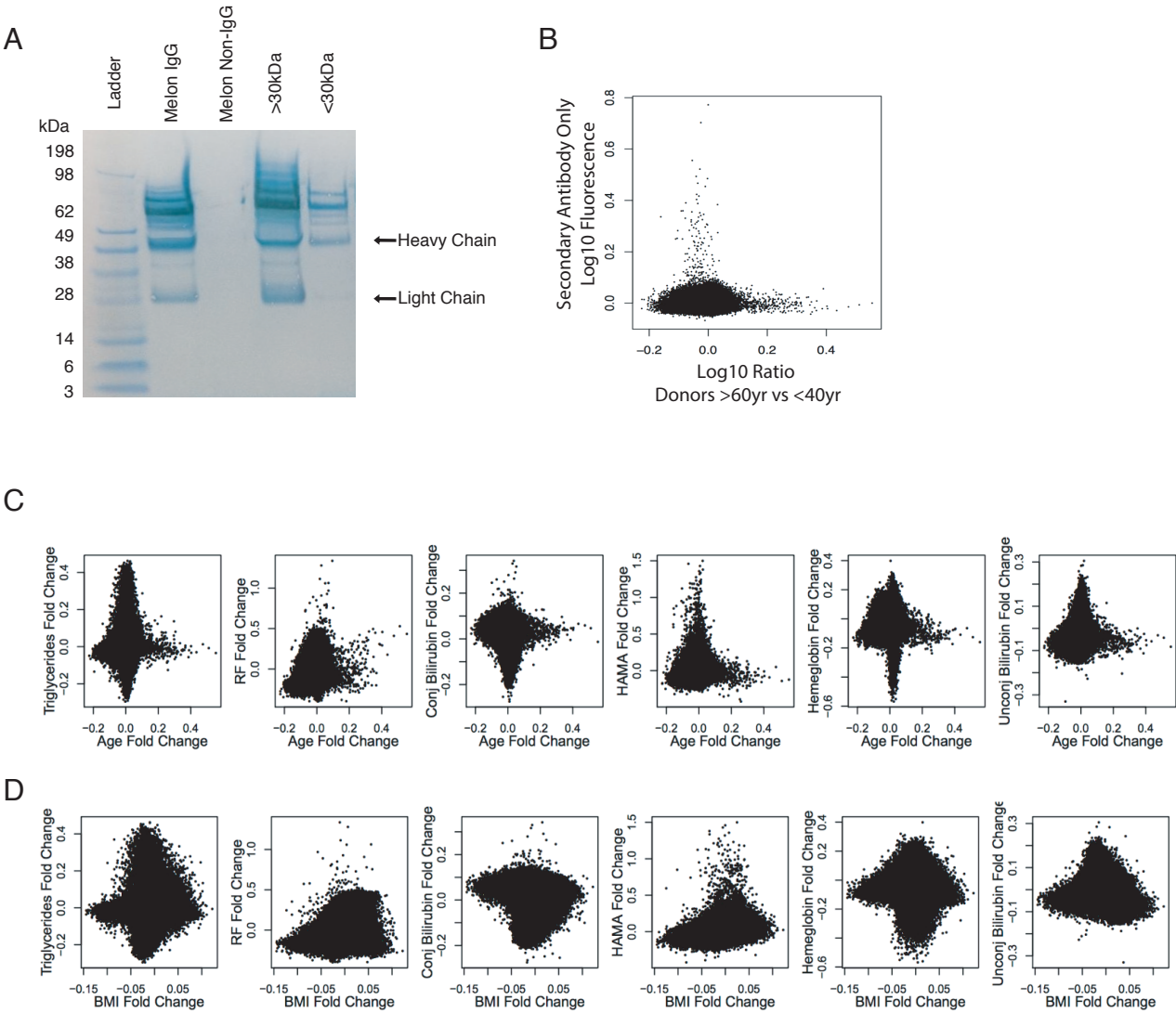

Supplement: Supplementary file 8 — Additional file 8: Figure S8. Additional control experiments suggest that age-associated antibody-peptide binding is driven by direct IgG binding. (A) Original image associated with Fig. 4b. In addition to filter columns, we also demonstrated IgG separation with Melon Gel purification; however, the Melon Gel purification assay reagents disrupted IgG-peptide array binding in the recombined fractions (the positive control) even though it produced superior IgG purification. It was thus excluded from downstream analysis. (B) The age-associated probes are not bound by anti-IgG secondary antibody, which is used for detecting IgG bound to peptides. Generic stickiness or peptides with similarity to IgG-Fc (which can partially bind secondary-antibody directly; y-axis) have minimal correlation with on age-association (x-axis). Each data point is a probe. (C-D) Interfering substances found in varying abundances in serum have limited impact on age-associated peptide probes. Serum from 4 healthy donors with and without an interfering substance was assayed by peptide microarray. Triglycerides, rheumatoid factor (RF), conjugated bilirubin, human anti-mouse antibody (HAMA), hemoglobin, and unconjugated bilirubin at a single high concentration (Methods). (C) The log10 ratio of serum with and without interfering substance (y-axis) is compared to the log10 ratio of serum from older (> 60 years) and younger (< 40 years) donors (x-axis). The only interfering substance that shows similar impact on peptides is RF, which is not statistically significant (p < 0.06, enrichment ratio of 1.9) as calculated by Fisher’s exact test (cutoffs were age log10 ratio > 0.25 and RF log10 ratio > 0.05; other cutoffs provided similar values). Pearson’s and Spearman correlation were not near significance, r = 0.06, rho = 0.03 (p < 0.54 and p < 0.69, respectively). (D) Similar to (C), except x-axis now shows the log10 ratio of serum from higher BMI (> 30) vs lower BMI (< 25). No interferants achieved statis [file 12979_2020_193_MOESM8_ESM.pdf]
