# Supplementary material for: Age-associated changes in the circulating human antibody repertoire are upregulated in autoimmunity
Source: Immun Ageing. 2020 Oct 6;17:28. doi: 10.1186/s12979-020-00193-x (PMC7539520; doi:10.1186/s12979-020-00193-x)
Supplement: Supplementary file 14 — Additional file 14: Table S6. Mapping from self-reported ethnicities to grouped ethnicities. [file 12979_2020_193_MOESM14_ESM.docx]

Supp Table S6. Mapping from self-report ethnicities to grouped ethnicities. Groupings aggregate samples based on broad ethnic backgrounds.

| Ethnicity Group | Self-Reported Ethnicity |
| --- | --- |
| White | “Non-Hispanic White” |
| Black | “Non-Hispanic Black” |
| Latino | “Other Hispanic/Spanish Some other race”, “Other Hispanic/Spanish White”, “Puerto Rican Some other race”, “Cuban White”, “Cuban Some other race”, “Mexican White”, “Mexican Some other race”. |
| Asian | “Non-Hispanic Chinese”, “Non-Hispanic Filipino”, “Non-Hispanic Vietnamese”, “Non-Hispanic Other Pacific Islander”, “Non-Hispanic Other Asian”, “Non-Hispanic Korean”, “Non-Hispanic Japanese”, “Non-Hispanic Asian Indian” |
| Other/Mixed | All others |
