# Supplementary material for: Age-associated changes in the circulating human antibody repertoire are upregulated in autoimmunity
Source: Immun Ageing. 2020 Oct 6;17:28. doi: 10.1186/s12979-020-00193-x (PMC7539520; doi:10.1186/s12979-020-00193-x)
Supplement: Supplementary file 15 — Additional file 15: Appendix [49–52]. [file 12979_2020_193_MOESM15_ESM.pdf]

SUPPLEMENTAL MATERIAL

The di-serine and other age-associated antibody-peptide binding is not impacted by exogenous spiking of endogenous age-associated interferants

To test if antibody-peptide binding was impacted by typical interferants, we used serum from four healthy donors with and without an interfering substance. We spiked interferants at a single high concentration: triglycerides, rheumatoid factor (RF), conjugated bilirubin, human anti-mouse antibody, hemoglobin, and unconjugated bilirubin (Methods). We then compared the log10 ratios of intensities measured with and without individual interferants to determine the impact the interferant had on peptide-array binding. Interestingly, we found that RF had an impact, though weak, on age-associated binding ($p < 0.06$, enrichment ratio of ~1.9). We examined a crystal structure of RF binding to its natural antigen, the antibody Fc region (1, 2). We observed that the heavy chain contains multiple di-serine and 'SSV' motifs ; however, most of these motifs were in the antibody Fab, not Fc, region and those in Fc were (1) not bound by RF in published crystal structures (PDB: 1ADQ) and (2) previous reports indicate that RF-shared epitope across RA patients and healthy donors does not include the di-serine motifs (3-6). Furthermore, murine immunization by human IgG resulted in similar epitopes as found in RA-patient rheumatoid factor, which also did not include binding to serine motifs. Additionally, the secondary antibody used, which binds to IgG Fc, was not associated with age-associated peptide features (Figure 4S1B), which indicates that generic serum antibody reactivity to IgG Fc is not associated with age.

Cytokine concentrations do not improve prediction of chronological age

We next assessed the ability to predict age from antibody binding and cytokine concentration data. Cytokines levels were measured by Luminex assay and quantified using the manufacturer provided protocol and software (Life Technologies Corp., Carlsbad, CA). A custom cytokine panel was used to measure CD40L, EGF, eotaxin (CCL11), GM-CSF, IFN-alpha, IFN-gamma, IL-1 beta, IL-1RA, IL-2R, IL-6, IP-10

(CSCL10), MCP-1 (CCL2), TNF-alpha, TNF-RI and TNF-RII. CRO and ACDC levels were measured separately. Four of the cytokines tested, IP10, eotaxin, sIL2Ra, and sTNFR1 were positively correlated with age while sCD40L was negatively correlated with age (Figure 3S4A). Two cytokines, sIL1Ra and CRP positively correlated with BMI (Figure 3S4B).

Given the prominent association of chronic inflammation biomarkers with age and BMI, we sought to understand if these markers could improve the antibody binding-based regression model. Binding and cytokine data were normalized by mean-centering after taking the log transform (Methods). To avoid explicit feature filtering, we used machine learning regression methods that encouraged sparsity to limit model complexity (Methods). We considered a number of machine learning models; however, we ultimately performed chronological age regression with a model that was a weighted linear combination of normalized fluorescent intensities (Methods).

When we combine cytokines as independent variables with all antibody-binding peptide features, we find cytokines do not add predictive capacity (Figure 3S4C). Chronological age predictions by serum cytokine levels and antibody profile generally agree with a Pearson correlation of r = 0.35 indicating a related but independent prediction (Figure 3S4D). Furthermore, we find that if we first develop an antibody binding score and combine this with each cytokine (which dramatically reduces dimensionality) and use ridge regression or elastic net, there is no improvement of prediction on an independent test set (not shown).

As we lack a gold standard for humoral immunosenescence, we moved ahead with antibody binding rather than cytokine measurements. This is because the cytokine markers, even in aggregate, were significantly less predictive of chronological age. Furthermore, adding cytokine markers on top of antibody binding did not add any predictive value.

Thus, while cytokines are important measurements of innate immunity, we focused attention on antibody binding measurements via peptide microarrays to better understand immunosenescence in the adaptive immune response.

1. Corper AL, Sohi MK, Bonagura VR, Steinitz M, Jefferis R, Feinstein A, Beale D, Taussig MJ, Sutton BJ. Structure of human IgM rheumatoid factor Fab bound to its autoantigen IgG Fc reveals a novel topology of antibody-antigen interaction. Nat Struct Biol. 1997;4(5):374-81. PubMed PMID: 9145108.
2. Duquerroy S, Stura EA, Bressanelli S, Fabiane SM, Vaney MC, Beale D, Hamon M, Casali P, Rey FA, Sutton BJ, Taussig MJ. Crystal structure of a human autoimmune complex between IgM rheumatoid factor RF61 and IgG1 Fc reveals a novel epitope and evidence for affinity maturation. J Mol Biol. 2007;368(5):1321-31. Epub 2007/03/06. doi: 10.1016/j.jmb.2007.02.085. PubMed PMID: 17395205; PMCID: PMC4625532.
3. Bonagura VR, Agostino N, Børretzen M, Thompson KM, Natvig JB, Morrison SL. Mapping IgG epitopes bound by rheumatoid factors from immunized controls identifies disease-specific rheumatoid factors produced by patients with rheumatoid arthritis. J Immunol. 1998;160(5):2496-505. PubMed PMID: 9498795.
4. Van Esch WJ, Reparon-Schuijt CC, Hamstra HJ, Van Kooten C, Logtenberg T, Breedveld FC, Verweij CL. Human IgG Fc-binding phage antibodies constructed from synovial fluid CD38+ B cells of patients with rheumatoid arthritis show the imprints of an antigen-dependent process of somatic hypermutation and clonal selection. Clin Exp Immunol. 2003;131(2):364-76. doi: 10.1046/j.1365-2249.2003.02068.x. PubMed PMID: 12562401; PMCID: PMC1808634.
5. Prokunina L, Padyukov L, Bennet A, de Faire U, Wiman B, Prince J, Alfredsson L, Klareskog L, Alarcón-Riquelme M. Association of the PD-1.3A allele of the PDCD1 gene in patients with rheumatoid arthritis negative for rheumatoid factor and the shared epitope. Arthritis Rheum. 2004;50(6):1770-3. doi: 10.1002/art.20280. PubMed PMID: 15188352.
6. Westwood OM, Nelson PN, Hay FC. Rheumatoid factors: what's new? Rheumatology (Oxford). 2006;45(4):379-85. Epub 2006/01/17. doi: 10.1093/rheumatology/kei228. PubMed PMID: 16418203.
